# Supplementary material for: Spatio-temporal estimates of HIV risk group proportions for adolescent girls and young women across 13 priority countries in sub-Saharan Africa
Source: PLOS Glob Public Health. 2023 Apr 19;3(4):e0001731. doi: 10.1371/journal.pgph.0001731 (PMC10115274; doi:10.1371/journal.pgph.0001731)
Supplement: S2 Text — (PDF) [file pgph.0001731.s002.pdf]

Supplementary tables and figures for "Spatio-temporal estimates of HIV risk group proportions for adolescent girls and young women across 13 priority countries in sub-Saharan Africa"

Corresponding author: Adam Howes (ath19@ic.ac.uk)

Contents

The Global AIDS Strategy 2

Household survey data 3

Included surveys . . . 3
Spatial analysis levels . . . 5
Survey questions and risk group allocation . . . 6

Miscellaneous figures 8

Country-specific figures 10

Comparison of direct and modelled risk group estimates . . . 10
HIV prevalence . . . 17
HIV incidence . . . 31
Expected new infections reached . . . 45

The Global AIDS Strategy

Prioritisation strata	Criterion
Low	0.3-1.0% incidence and low-risk behaviour, or <0.3% incidence and high-risk behaviour
Moderate	1.0-3.0% incidence and low-risk behaviour, or 0.3-1.0% incidence and high-risk behaviour
High	1.0-3.0% incidence and high-risk behaviour
Very high	>3.0% incidence

Table A: Prioritisation strata according to HIV incidence in the general population and behavioural risk.

Intervention	Low	Moderate	High	Very High
Condoms and lube for those with non-regular partners(s) with unknown STI status and not on PrEP	50%	70%	95%	95%
STI screening and treatment	10%	10%	80%	80%
Access to PEP	-	-	50%	90%
PrEP use	-	5%	50%	50%
Economic empowerment	-	-	20%	20%

Table B: Commitments to be met for each intervention in terms of proportion of the prioritisation strata reached, where "-" represents no commitment.

Household survey data

Included surveys

	Type	Year	Transactional sex question	Sample size			
				15-19	20-24	25-29	Total
Botswana							
	BAIS	2013	✓	557	588	649	1794
Total				557	588	649	1794
Cameroon							
	DHS	2004	✗	2675	2207	1732	6614
	DHS	2011	✗	3588	3115	2655	9358
	PHIA	2017	✗	2620	2339	2259	7218
	DHS	2018	✓	3349	2463	2345	8157
Total				12232	10124	8991	31347
Kenya							
	DHS	2003	✗	1819	1709	1391	4919
	DHS	2008	✗	1767	1743	1419	4929
	DHS	2014	✗	2861	2534	2858	8253
Total				6447	5986	5668	18101
Lesotho							
	DHS	2004	✗	1761	1455	1026	4242
	DHS	2009	✗	1833	1543	1194	4570
	DHS	2014	✗	1537	1292	1067	3896
	PHIA	2017	✓	1156	1202	1054	3412
Total				6287	5492	4341	16120
Mozambique							
	AIS	2009	✗	1031	1106	987	3124
	DHS	2011	✗	2932	2299	2206	7437
	AIS	2015	✗	1552	1389	1080	4021
Total				5515	4794	4273	14582
Malawi							
	DHS	2000	✗	2914	2998	2358	8270
	DHS	2004	✗	2407	2823	2135	7365
	DHS	2010	✗	5031	4387	4309	13727
	DHS	2015	✓	5273	5094	3976	14343
	PHIA	2016	✓	1646	1934	1511	5091
Total				17271	17236	14289	48796
Namibia							
	DHS	2000	✗	1427	1313	1098	3838
	DHS	2006	✗	2203	1869	1544	5616
	DHS	2013	✗	1852	1709	1481	5042
	PHIA	2017	✓	1491	1525	1370	4386
Total				6973	6416	5493	18882
Eswatini							
	DHS	2006	✗	1265	1027	731	3023
	PHIA	2017	✗	1031	895	811	2737

Total				2296	1922	1542	5760
Tanzania							
	AIS	2003	✗	1466	1377	1270	4113
	AIS	2007	✗	2137	1676	1509	5322
	DHS	2010	✗	2221	1860	1613	5694
	AIS	2012	✗	2474	1923	1815	6212
	PHIA	2016	✓	2999	2845	2521	8365
Total				11297	9681	8728	29706
Uganda							
	DHS	2000	✗	1687	1541	1326	4554
	DHS	2006	✗	1948	1660	1404	5012
	AIS	2011	✗	2451	2164	1921	6536
	DHS	2011	✗	2025	1664	1614	5303
	DHS	2016	✓	4276	3782	3014	11072
	PHIA	2016	✗	3289	3059	2574	8922
Total				15676	13870	11853	41399
South Africa							
	DHS	2016	✓	1505	1408	1397	4310
Total				1505	1408	1397	4310
Zambia							
	DHS	2007	✗	1598	1405	1373	4376
	DHS	2013	✗	3685	3036	2789	9510
	PHIA	2016	✓	2120	2045	1619	5784
	DHS	2018	✓	3112	2687	2166	7965
Total				10515	9173	7947	27635
Zimbabwe							
	DHS	1999	✗	1467	1230	1011	3708
	DHS	2005	✗	2128	1943	1438	5509
	DHS	2010	✗	1963	1796	1679	5438
	DHS	2015	✓	2154	1777	1646	5577
	PHIA	2016	✓	2114	1817	1573	5504
Total				9826	8563	7347	25736
Total				106397	95253	82518	284168

Table C: All of the surveys that we used in our analysis and their sample sizes, disaggregated by respondent age.

Survey	Exclusion reason
MOZ2003DHS	No GPS coordinates available to place survey clusters within districts.
TZA2015DHS	Insufficient sexual behaviour questions.
UGA2004AIS	Unable to download region boundaries.
ZMB2002DHS	No GPS coordinates available to place survey clusters within districts.

Table D: All of that surveys that were excluded from our analysis.

Spatial analysis levels

Country	Number of areas	Analysis level
Botswana	27	3
Cameroon	58	2
Kenya	47	2
Lesotho	10	1
Mozambique	161	3
Malawi	33	5
Namibia	38	2
Eswatini	4	1
Tanzania	195	4
Uganda	136	3
South Africa	52	2
Zambia	116	2
Zimbabwe	63	2

Table E: The numer of areas and analysis levels for each country that were used in our analysis.

Survey questions and risk group allocation

Variable(s)	Description
`v501`	Current marital status of the respondent.
`v529`	Computed time since last sexual intercourse.
`v531`	Age at first sexual intercourse–imputed.
`v766b`	Number of sexual partners during the last 12 months (including husband).
`v767[a, b, c]`	Relationship with last three sexual partners. Options are: spouse, boyfriend not living with respondent, other friend, casual acquaintance, relative, commercial sex worker, live-in partner, other.
`v791a`	Had sex in return for gifts, cash or anything else in the past 12 months. Asked only to women 15-24 who are not in a union.

Table F: AIS, BAIS and DHS survey questions.

Variable(s)	Description
`part12monum`	Number of sexual partners during the last 12 months (including husband).
`part12modkr`	Reason for leaving `part12monum` blank.
`partlivew[1, 2, 3]`	Does the person you had sex with live in this household?
`partrelation[1, 2, 3]`	Relationship with last three sexual partners. Options are: husband, live-in partner, partner (not living with), ex-spouse/partner, friend/acquaintance, sex worker, sex worker client, stranger, other, don't know, refused.
`sellsx12mo`	Had sex for money and/or gifts in the last 12 months.
`buysx12mo`	Paid money or given gifts for sex in the last 12 months.

Table G: PHIA survey questions.

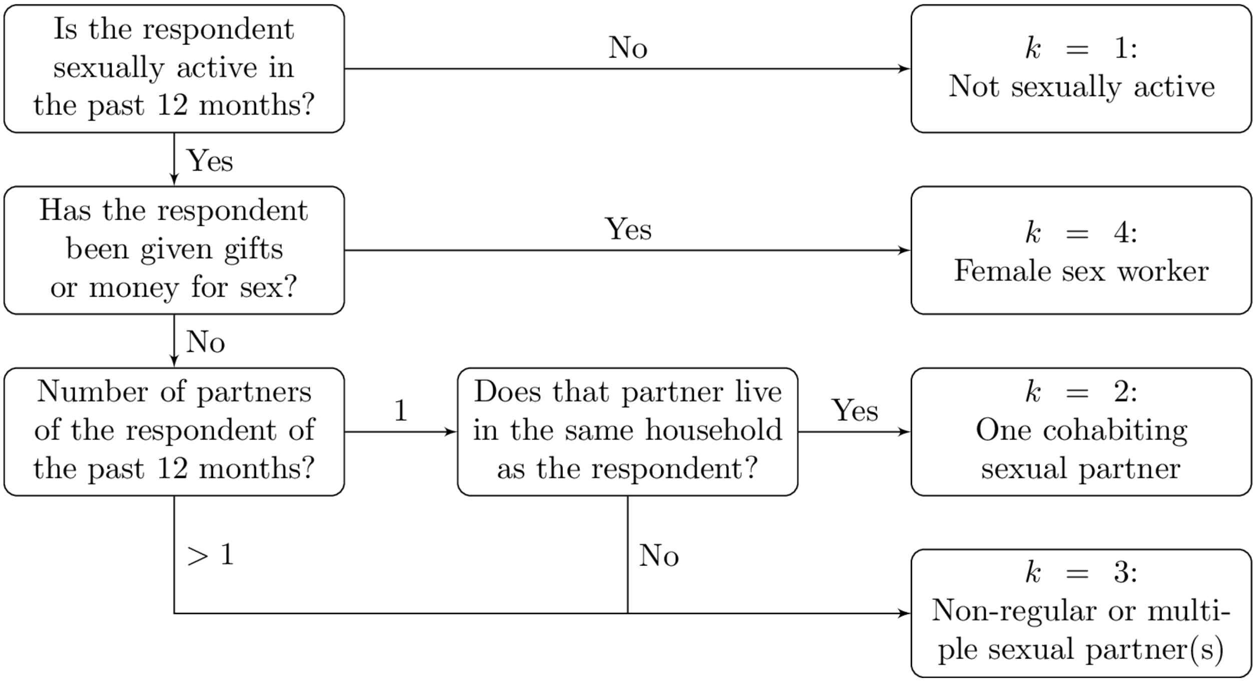

Figure A: Flowchart describing allocation of respondents to risk groups.

Miscellaneous figures

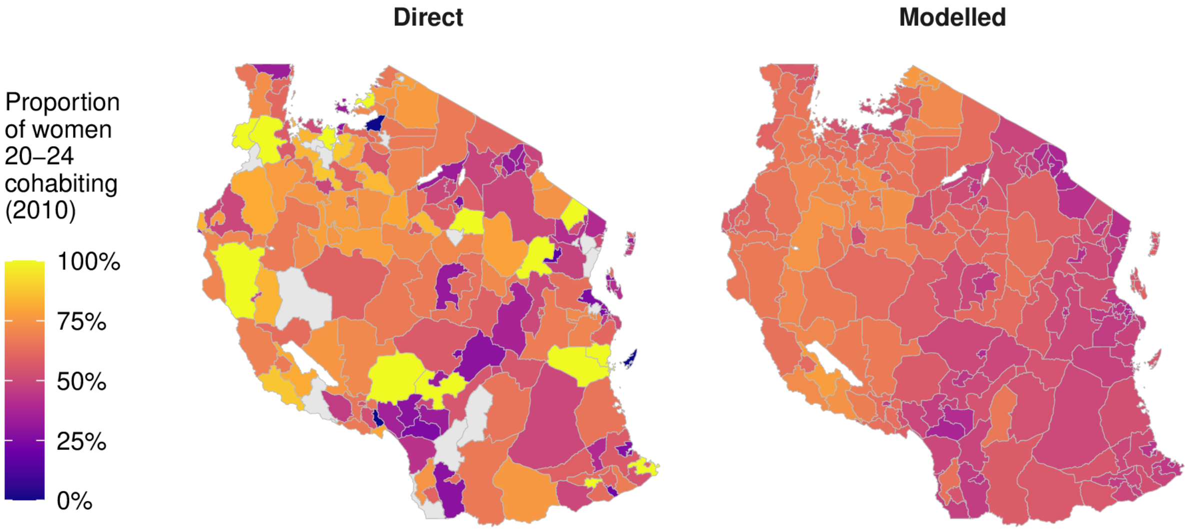

Figure B: Illustration of our model results for AGYW 20-24 in Tanzania in 2010 in the cohabiting risk group. Compared to the direct survey results, our spatio-temporally smoothed estimates more plausibly represent district-level heterogeneity, as well as imputing any districts with missing data.

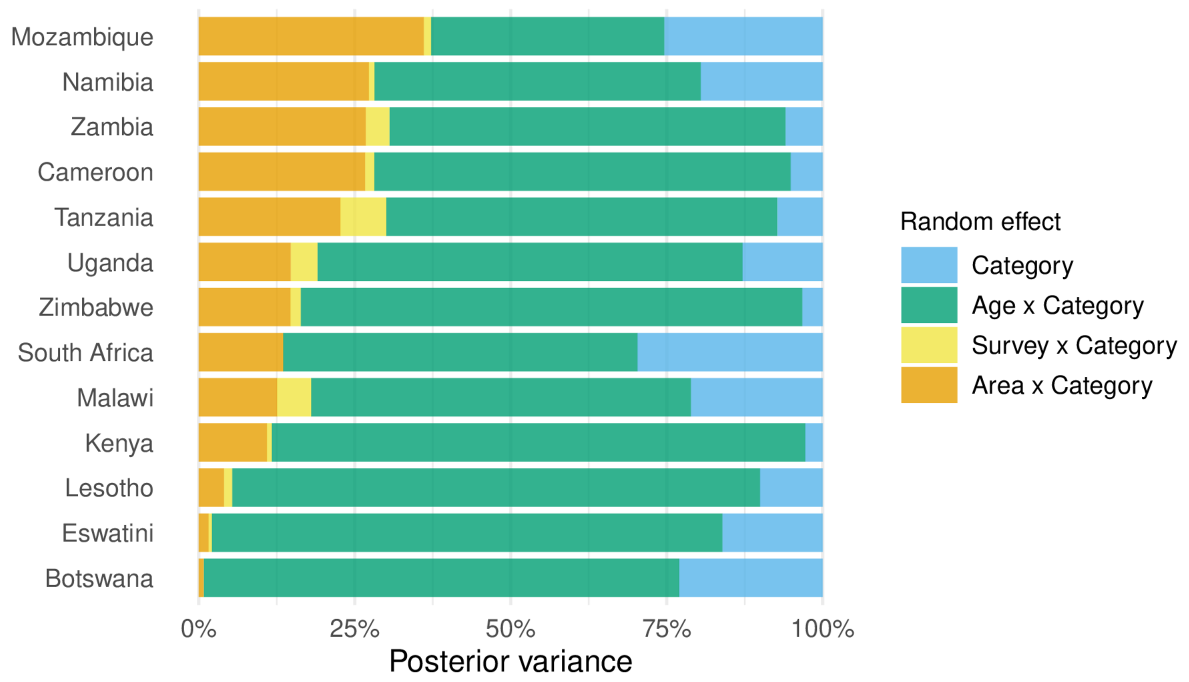

Figure C: Proportion of variance explained by each random effect (Sobol' indices) when the multinomial regression model is fit to each country individually. In this setting, country-category random effects are not included in the model and year-category random effects are replaced by survey-category random effects (for countries with surveys in multiple years). Countries are ordered by the proportion of their variance which is explained by the area-category random effects.

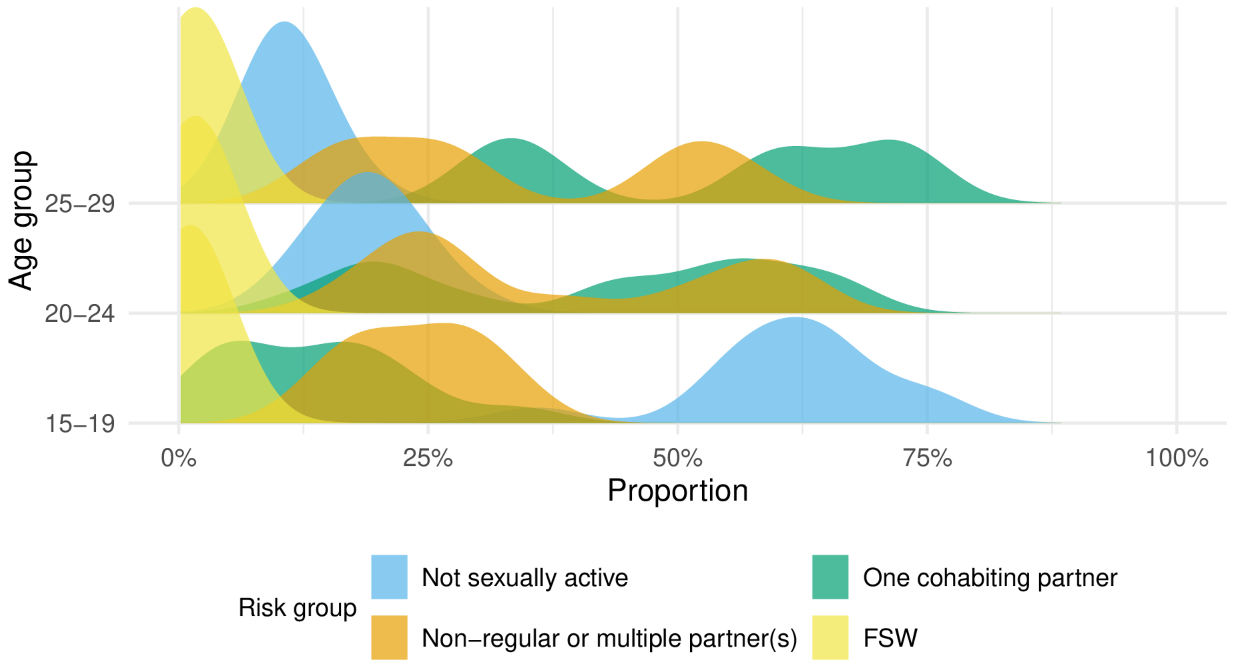

Figure D: The posterior density of national-level risk group proportions by age, illustrating the bi-modality of the cohabiting partner and non-regular and multiple partner(s) risk groups.

Country-specific figures

Comparison of direct and modelled risk group estimates

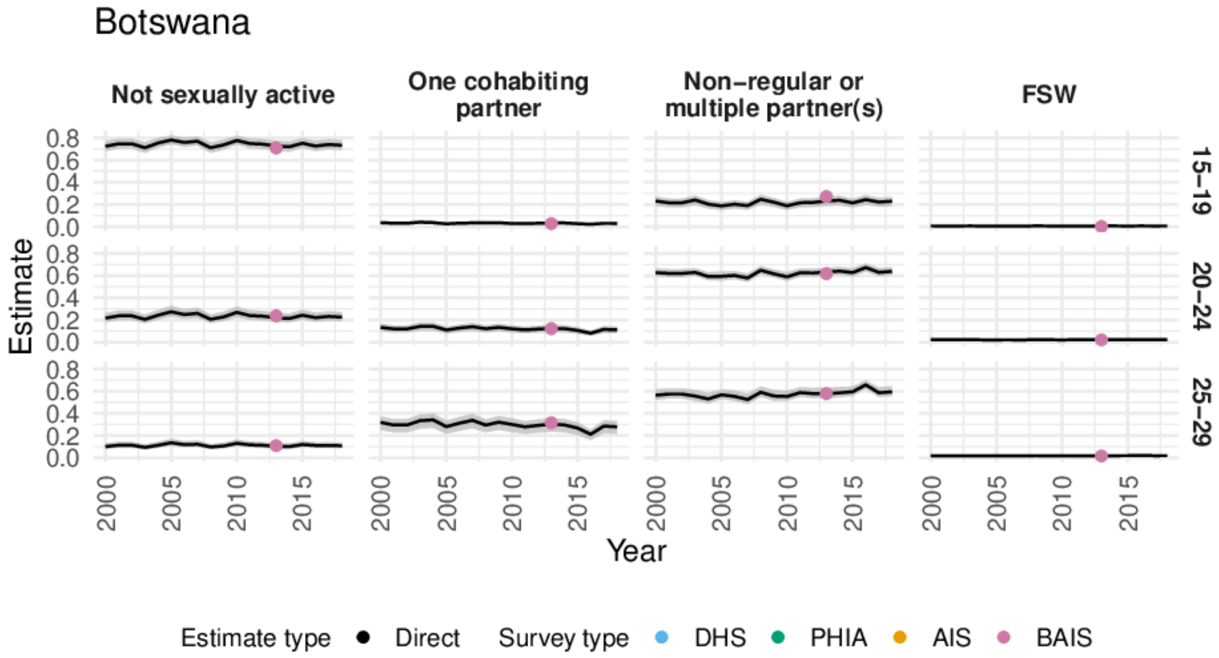

Figure E: Comparison of modelled and direct national-level estimates in 1999-2018 in Botswana. Estimates are described as "partially direct" when there are no surveys containing a transactional sex question in a country-age-group and we instead used modelled logistic regression estimates to differentiate the direct estimates.

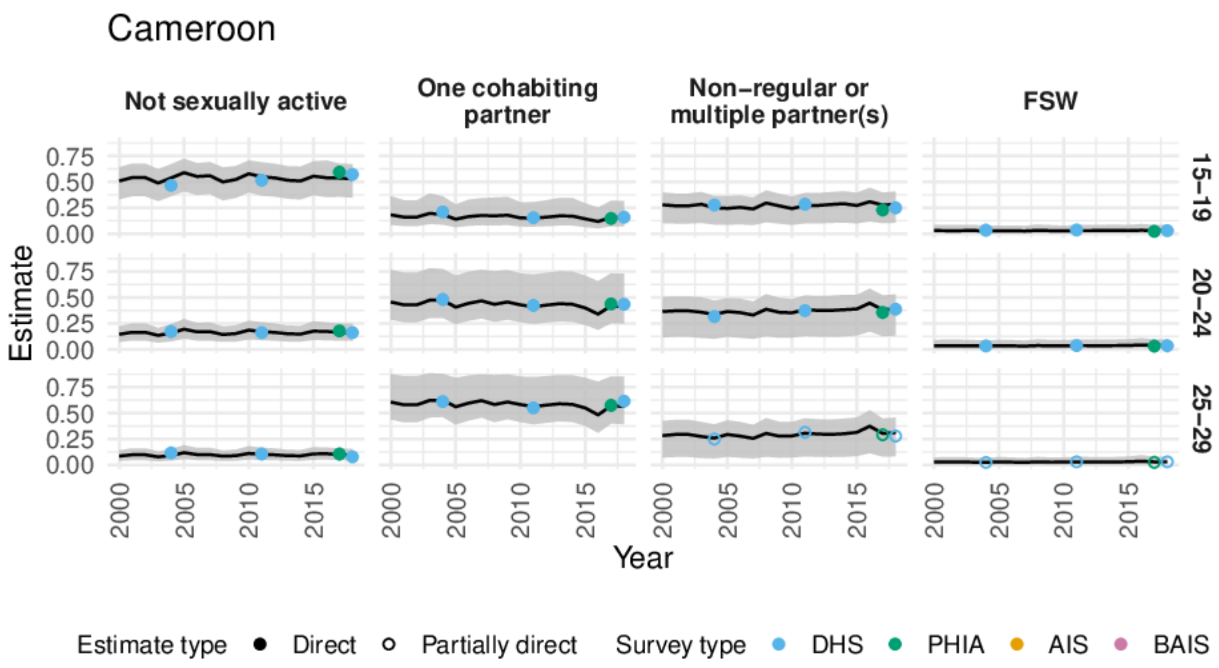

Figure F: Comparison of modelled and direct national-level estimates in 1999-2018 in Cameroon. Estimates are described as "partially direct" when there are no surveys containing a transactional sex question in a country-age-group and we instead used modelled logistic regression estimates to differentiate the direct estimates.

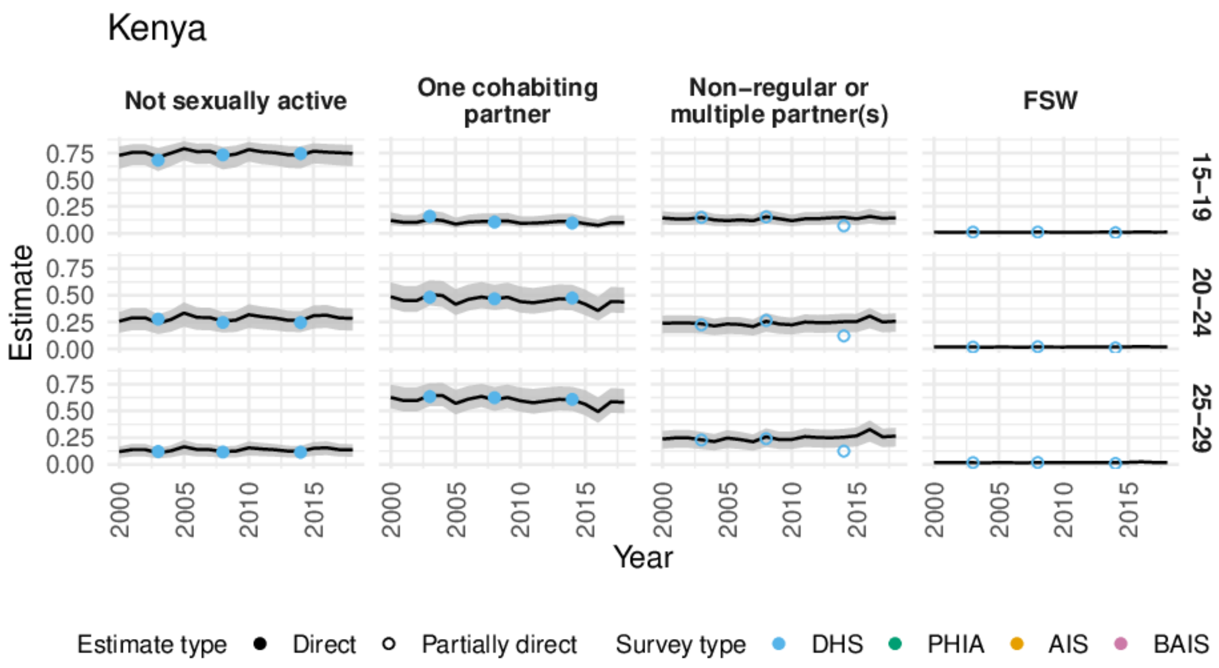

Figure G: Comparison of modelled and direct national-level estimates in 1999-2018 in Kenya. Estimates are described as "partially direct" when there are no surveys containing a transactional sex question in a country-age-group and we instead used modelled logistic regression estimates to differentiate the direct estimates.

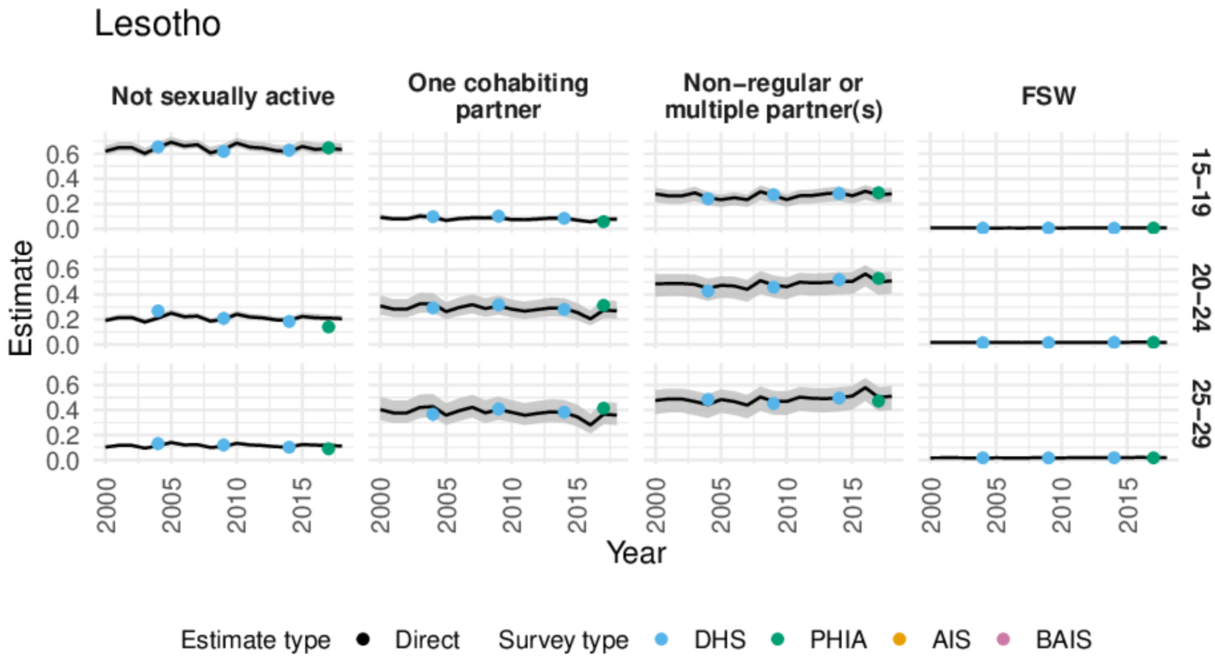

Figure H: Comparison of modelled and direct national-level estimates in 1999-2018 in Lesotho. Estimates are described as "partially direct" when there are no surveys containing a transactional sex question in a country-age-group and we instead used modelled logistic regression estimates to differentiate the direct estimates.

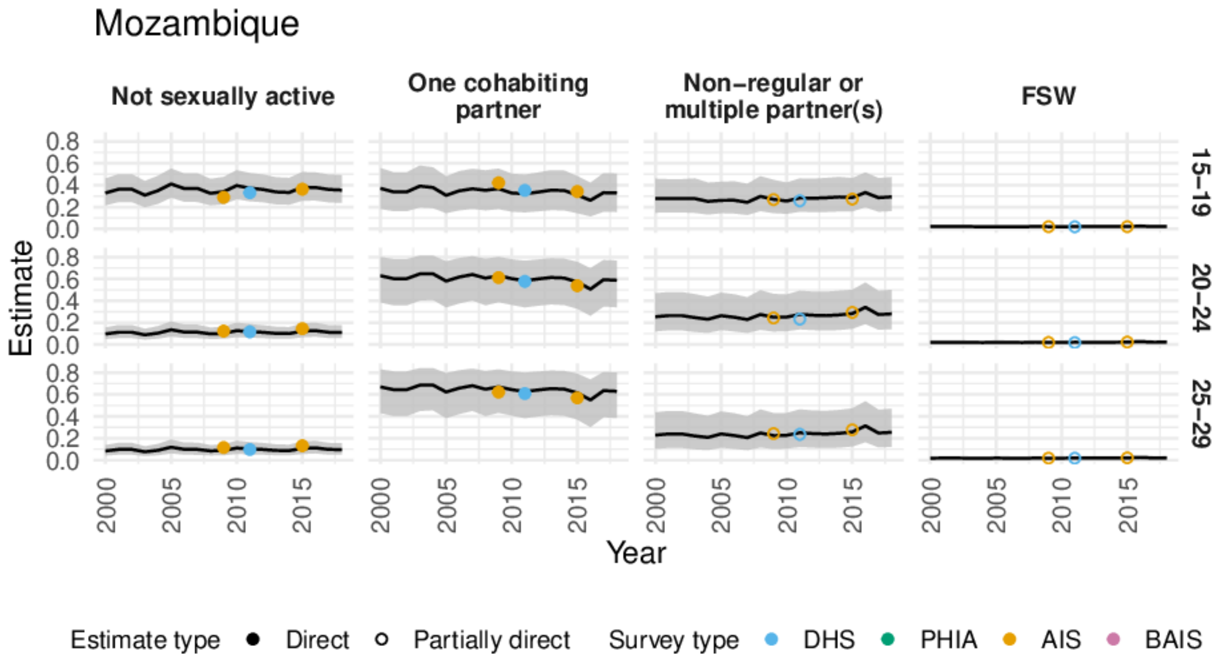

Figure I: Comparison of modelled and direct national-level estimates in 1999-2018 in Mozambique. Estimates are described as "partially direct" when there are no surveys containing a transactional sex question in a country-age-group and we instead used modelled logistic regression estimates to differentiate the direct estimates.

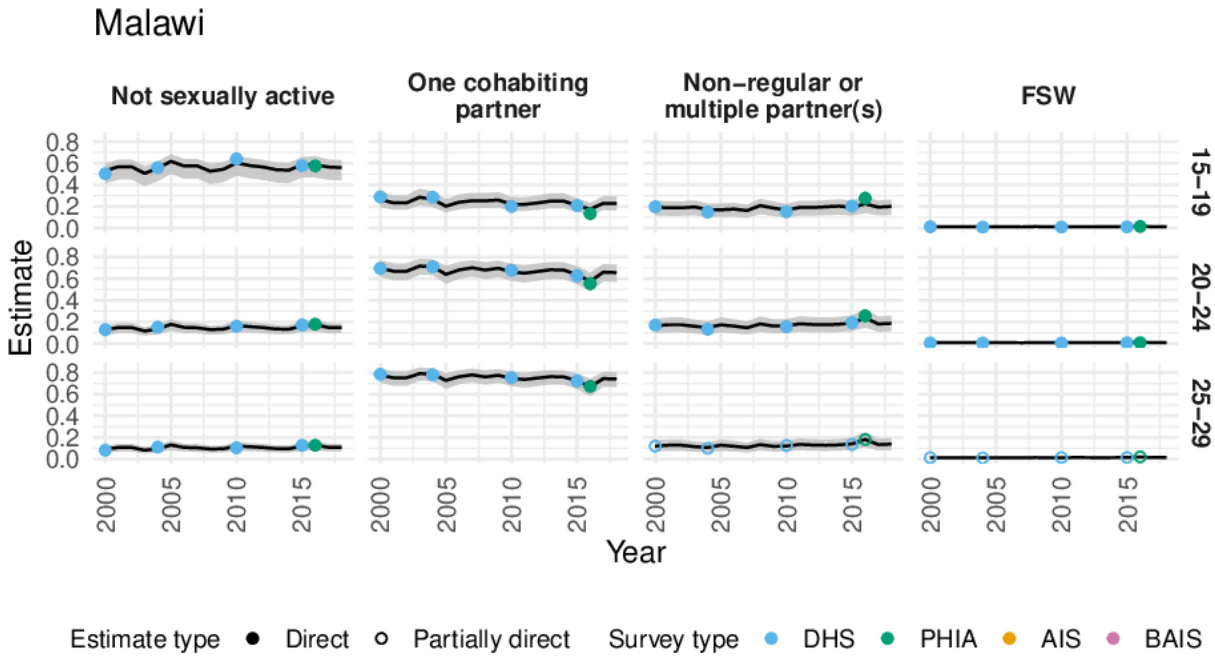

Figure J: Comparison of modelled and direct national-level estimates in 1999-2018 in Malawi. Estimates are described as "partially direct" when there are no surveys containing a transactional sex question in a country-age-group and we instead used modelled logistic regression estimates to differentiate the direct estimates.

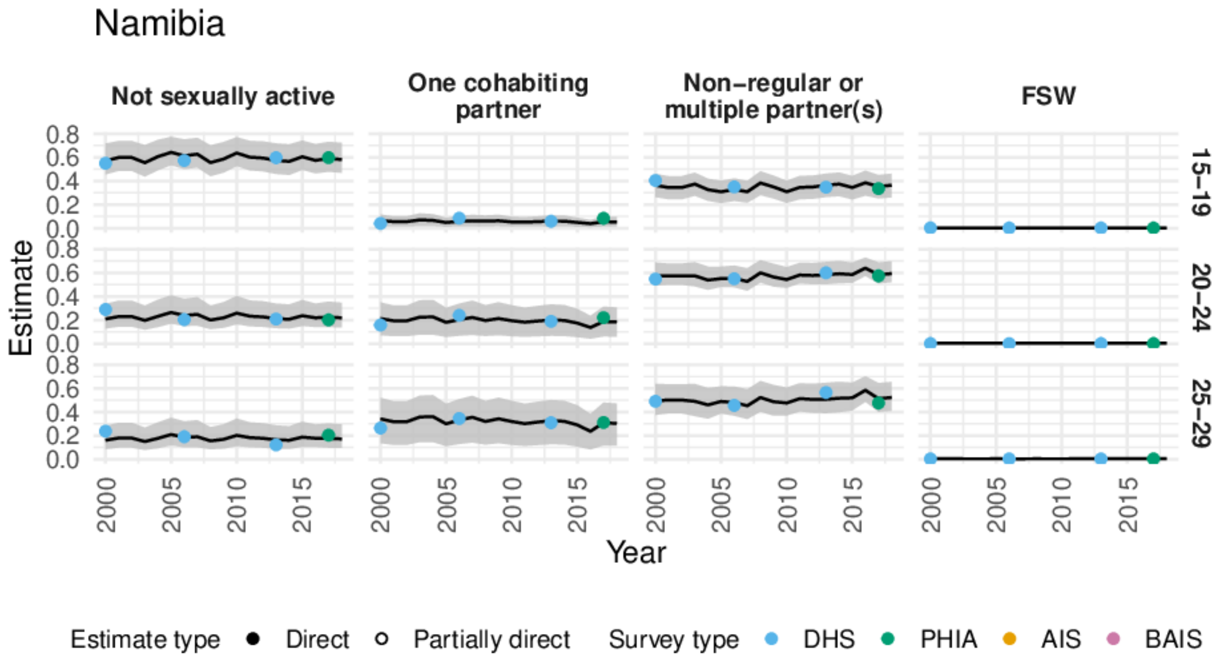

Figure K: Comparison of modelled and direct national-level estimates in 1999-2018 in Namibia. Estimates are described as "partially direct" when there are no surveys containing a transactional sex question in a country-age-group and we instead used modelled logistic regression estimates to differentiate the direct estimates.

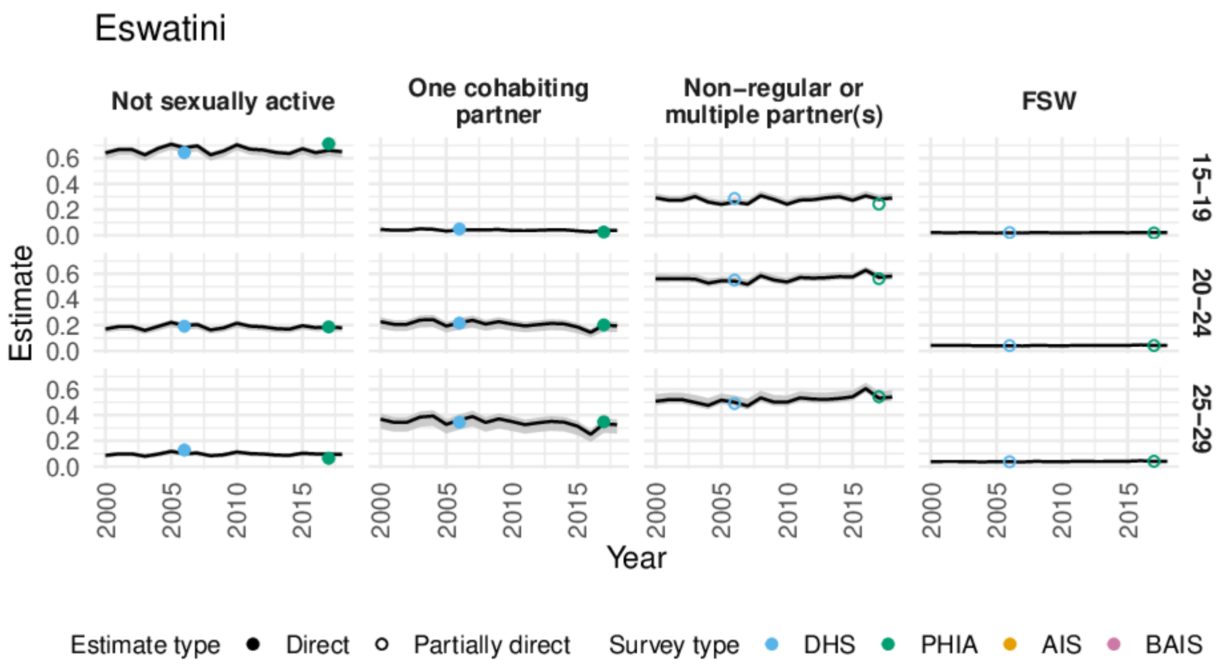

Figure L: Comparison of modelled and direct national-level estimates in 1999-2018 in Eswatini. Estimates are described as "partially direct" when there are no surveys containing a transactional sex question in a country-age-group and we instead used modelled logistic regression estimates to differentiate the direct estimates.

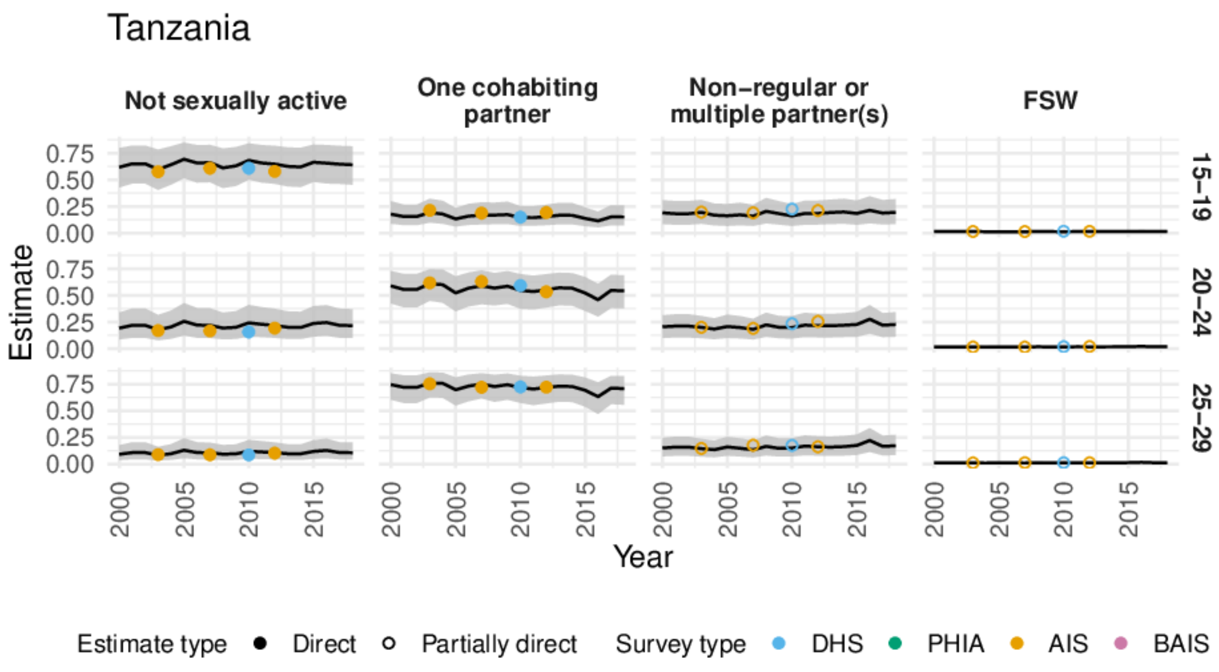

Figure M: Comparison of modelled and direct national-level estimates in 1999-2018 in Tanzania. Estimates are described as "partially direct" when there are no surveys containing a transactional sex question in a country-age-group and we instead used modelled logistic regression estimates to differentiate the direct estimates.

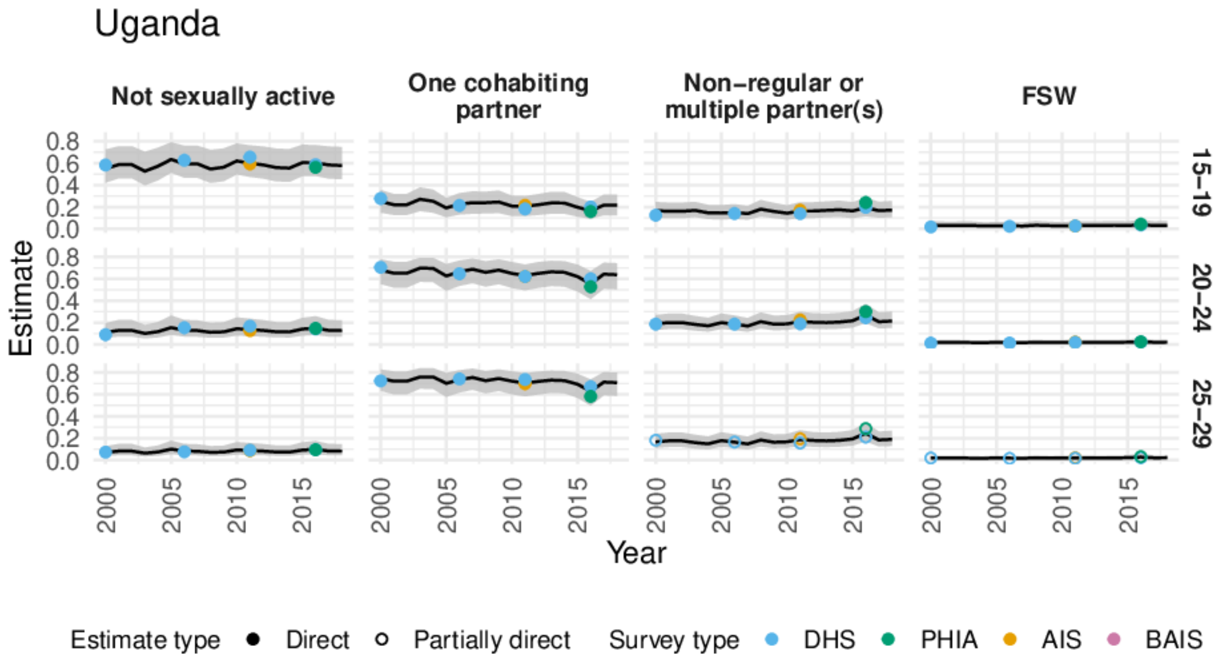

Figure N: Comparison of modelled and direct national-level estimates in 1999-2018 in Uganda. Estimates are described as "partially direct" when there are no surveys containing a transactional sex question in a country-age-group and we instead used modelled logistic regression estimates to differentiate the direct estimates.

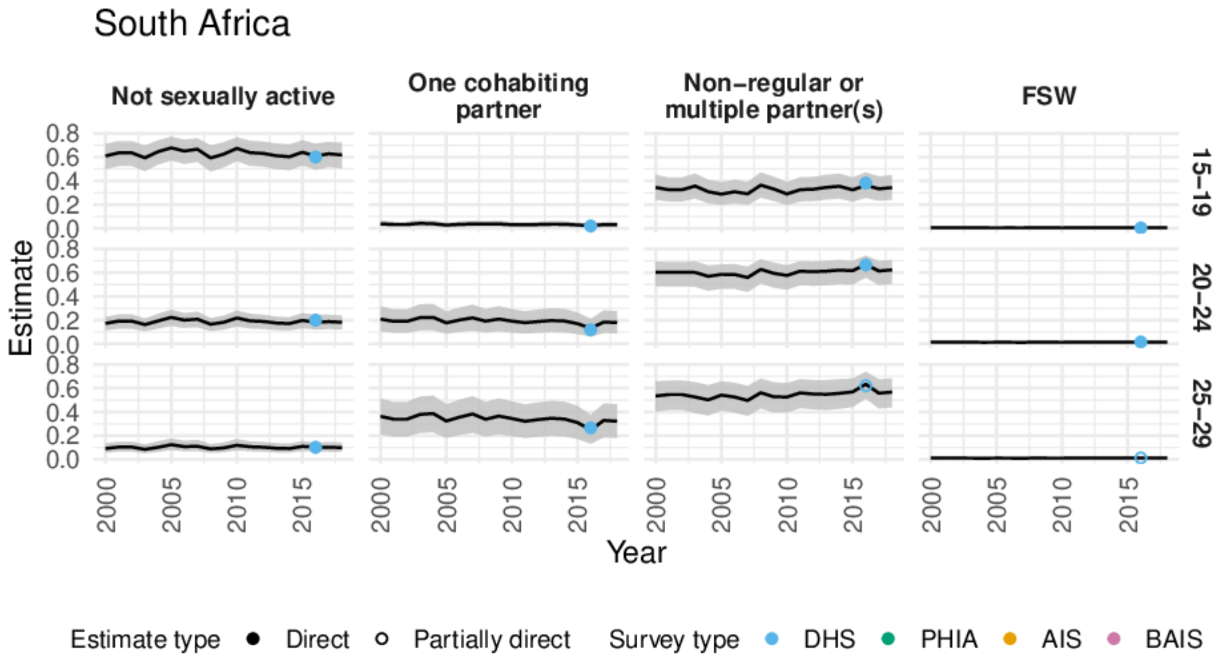

Figure O: Comparison of modelled and direct national-level estimates in 1999-2018 in South Africa. Estimates are described as "partially direct" when there are no surveys containing a transactional sex question in a country-age-group and we instead used modelled logistic regression estimates to differentiate the direct estimates.

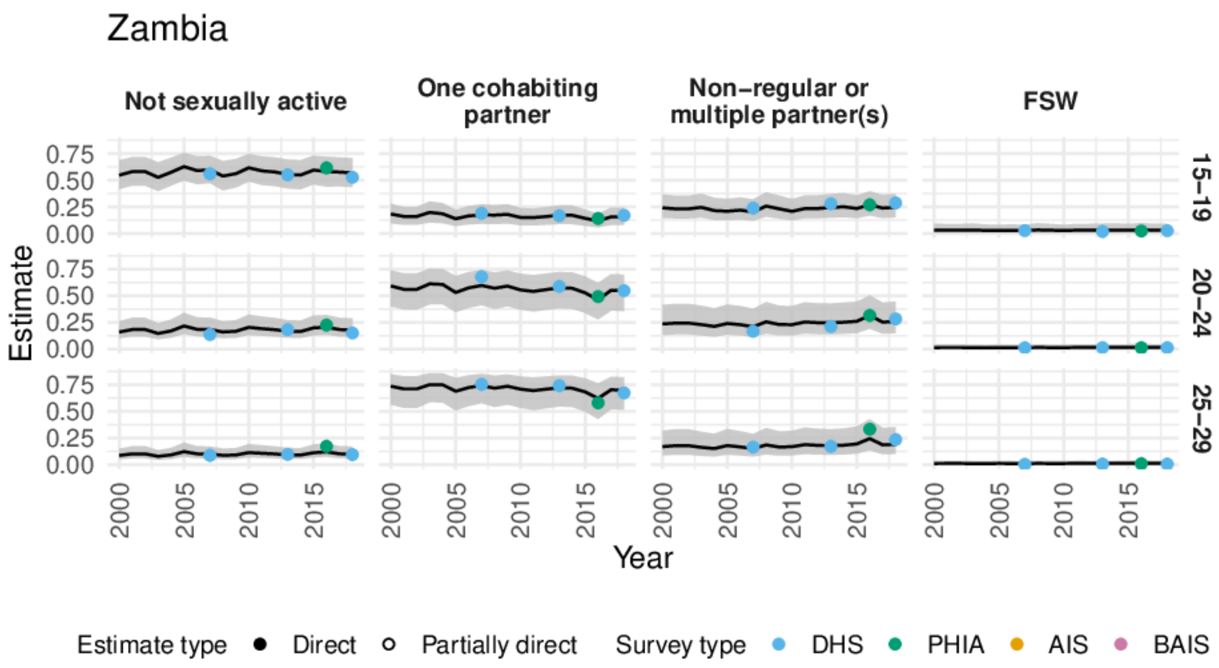

Figure P: Comparison of modelled and direct national-level estimates in 1999-2018 in Zambia. Estimates are described as "partially direct" when there are no surveys containing a transactional sex question in a country-age-group and we instead used modelled logistic regression estimates to differentiate the direct estimates.

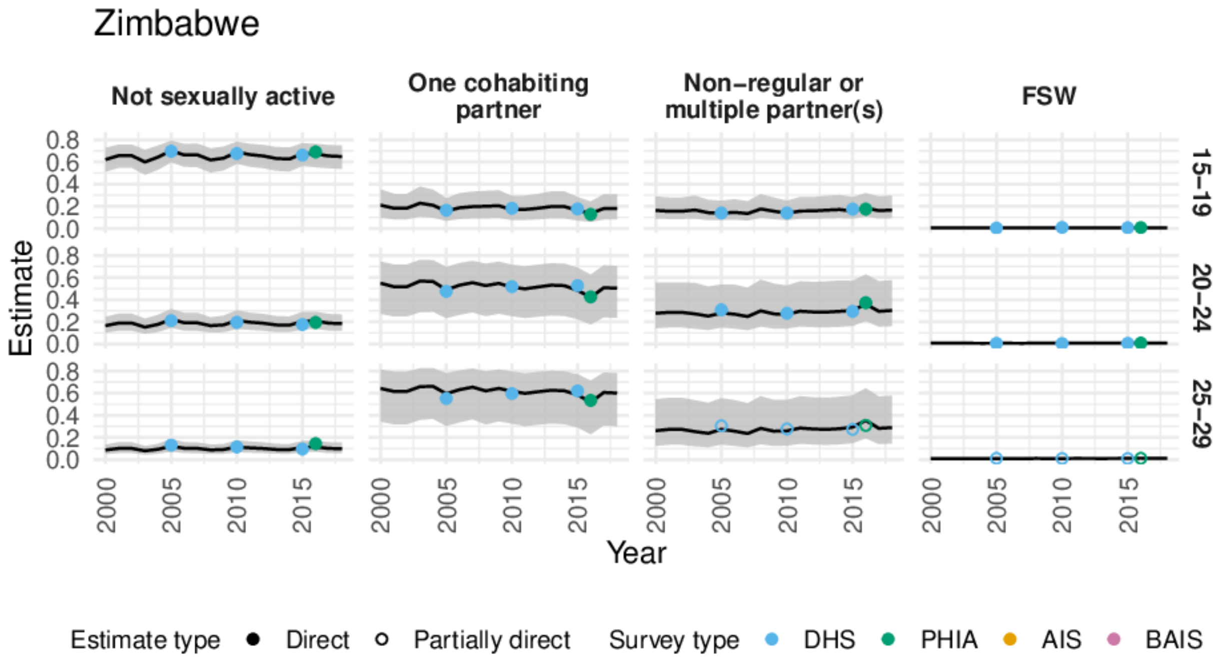

Figure Q: Comparison of modelled and direct national-level estimates in 1999-2018 in Zimbabwe. Estimates are described as "partially direct" when there are no surveys containing a transactional sex question in a country-age-group and we instead used modelled logistic regression estimates to differentiate the direct estimates.

HIV prevalence

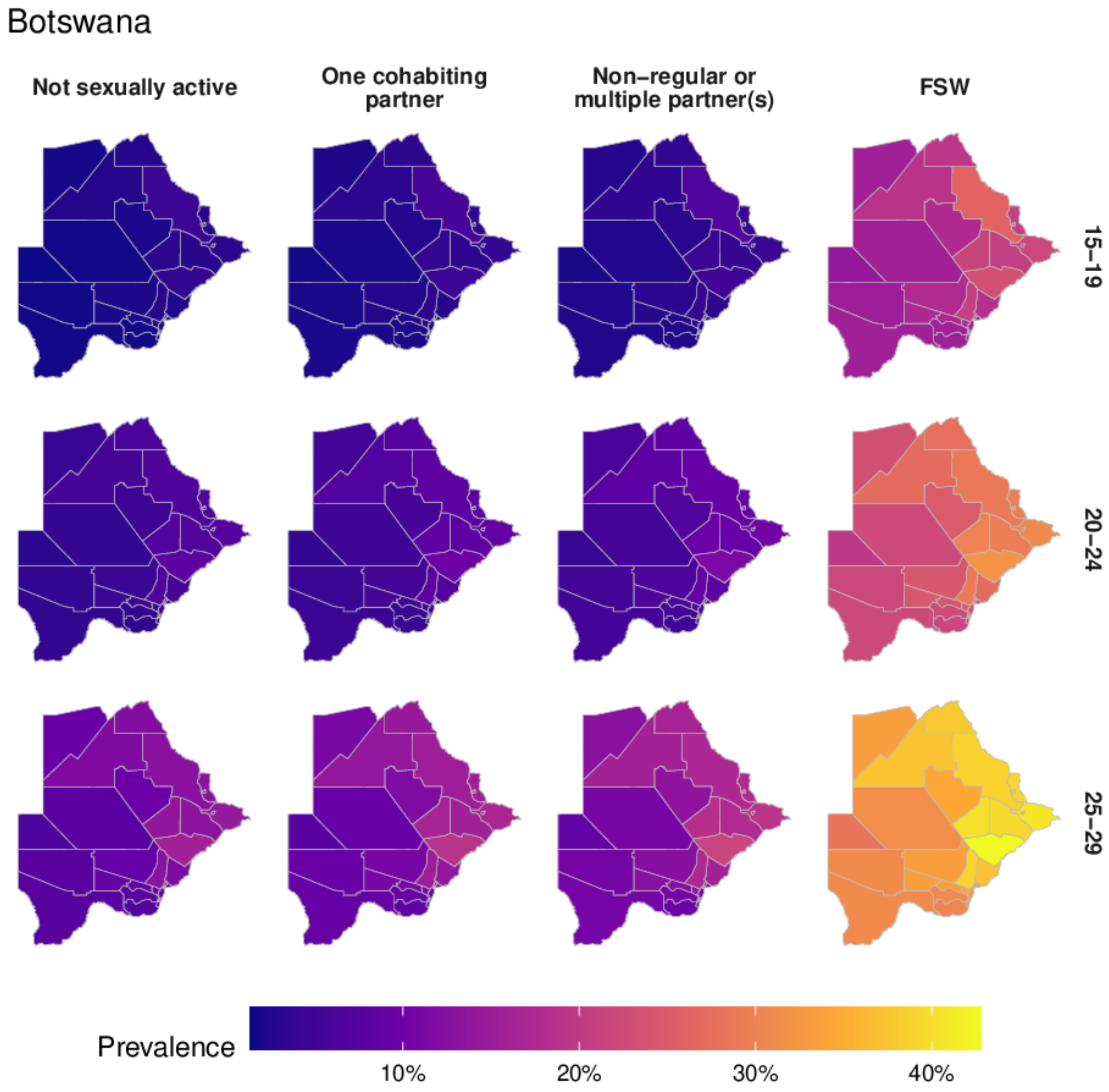

Figure R: District-level HIV prevalence for each of the risk groups in 2018 in Botswana.

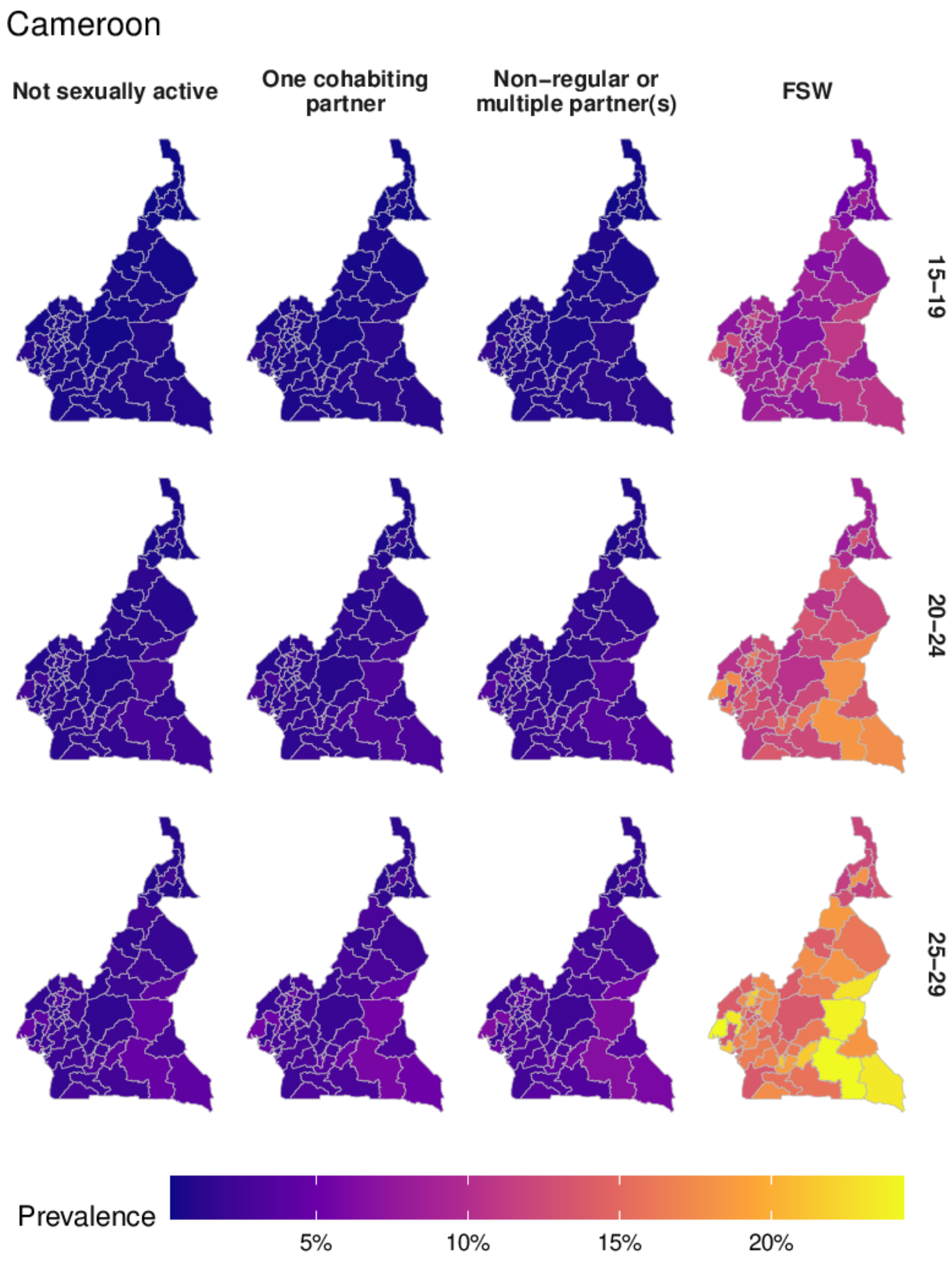

Figure S: District-level HIV prevalence for each of the risk groups in 2018 in Cameroon.

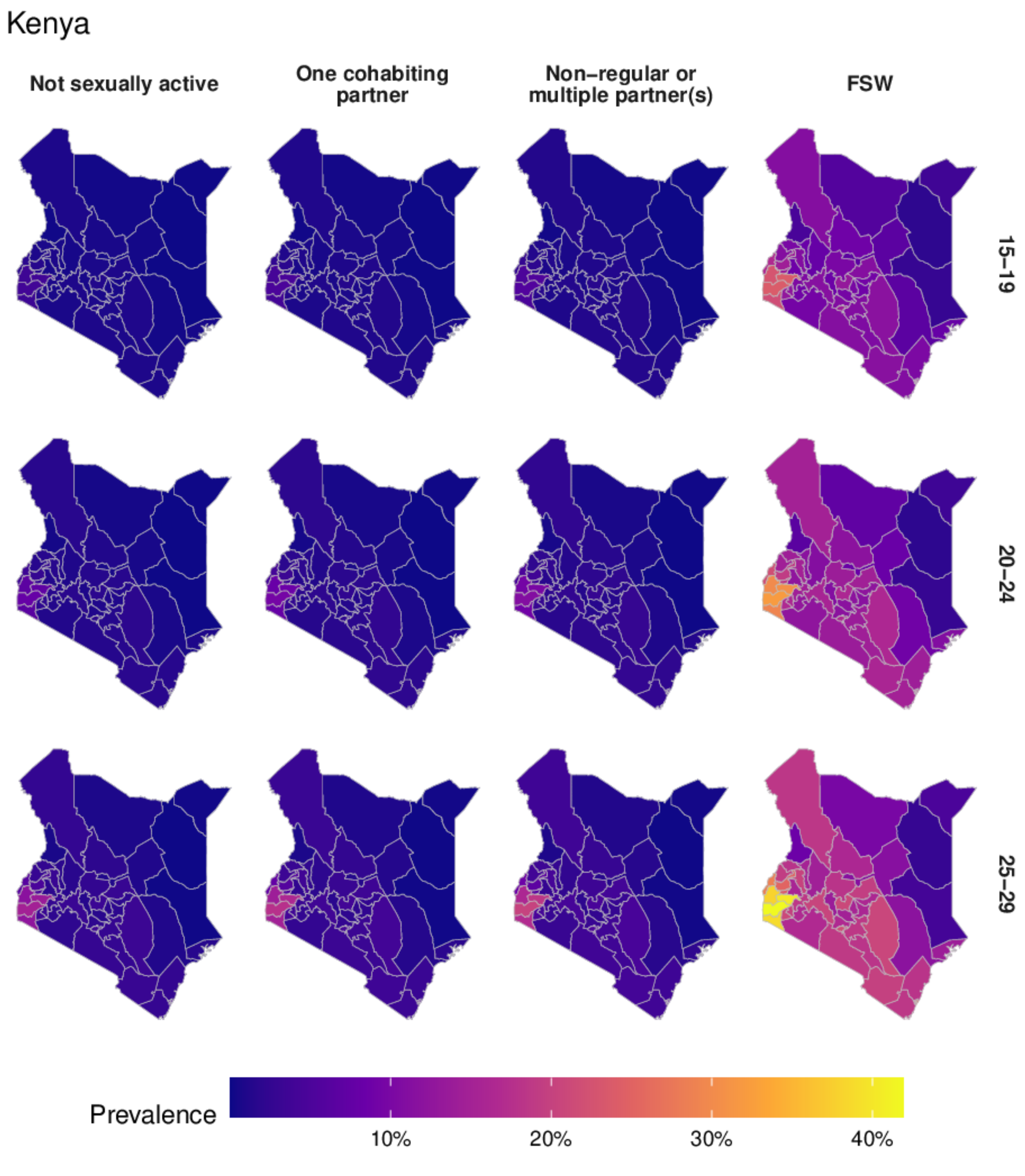

Figure T: District-level HIV prevalence for each of the risk groups in 2018 in Kenya.

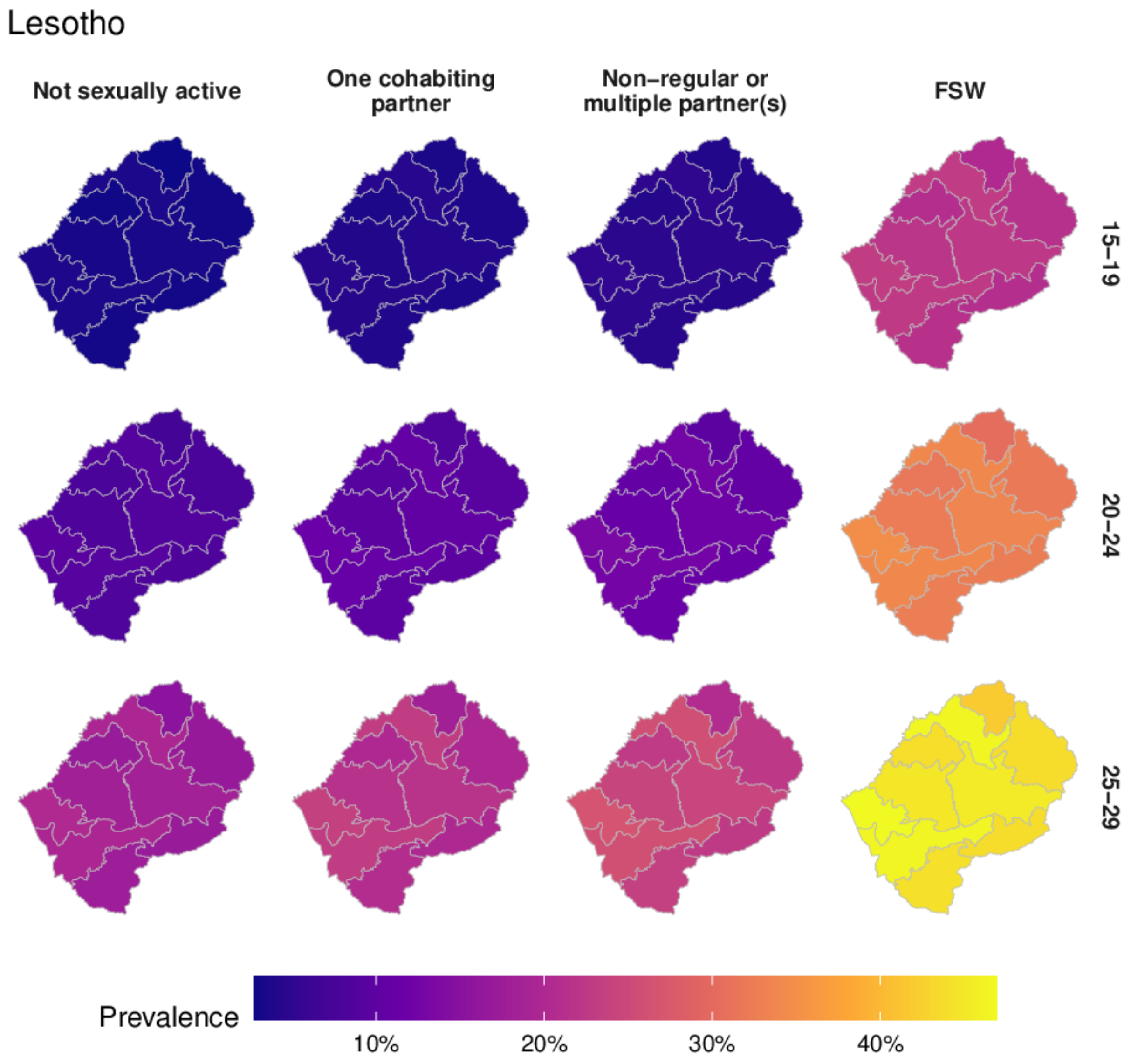

Figure U: District-level HIV prevalence for each of the risk groups in 2018 in Lesotho.

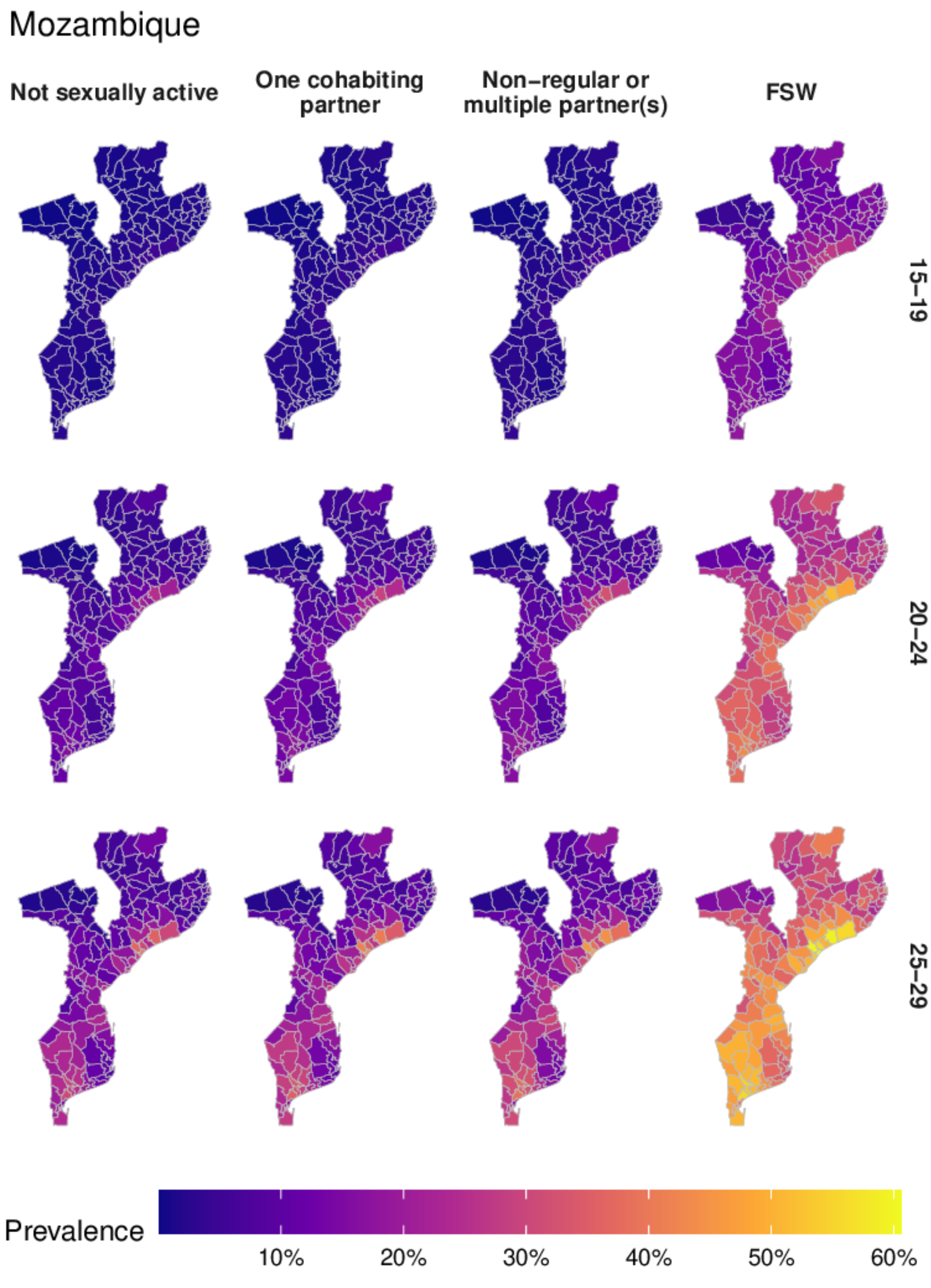

Figure V: District-level HIV prevalence for each of the risk groups in 2018 in Mozambique.

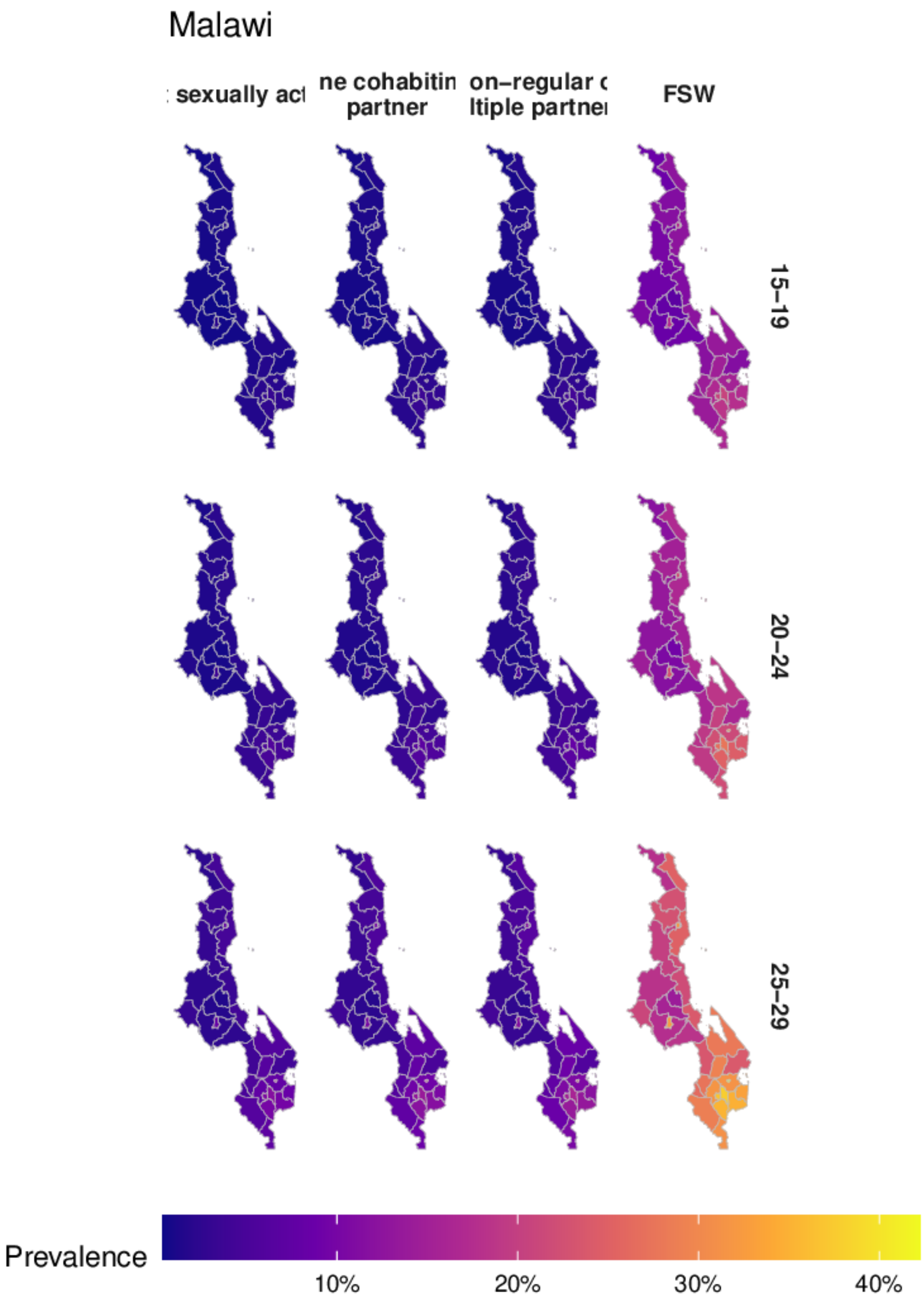

Figure W: District-level HIV prevalence for each of the risk groups in 2018 in Malawi.

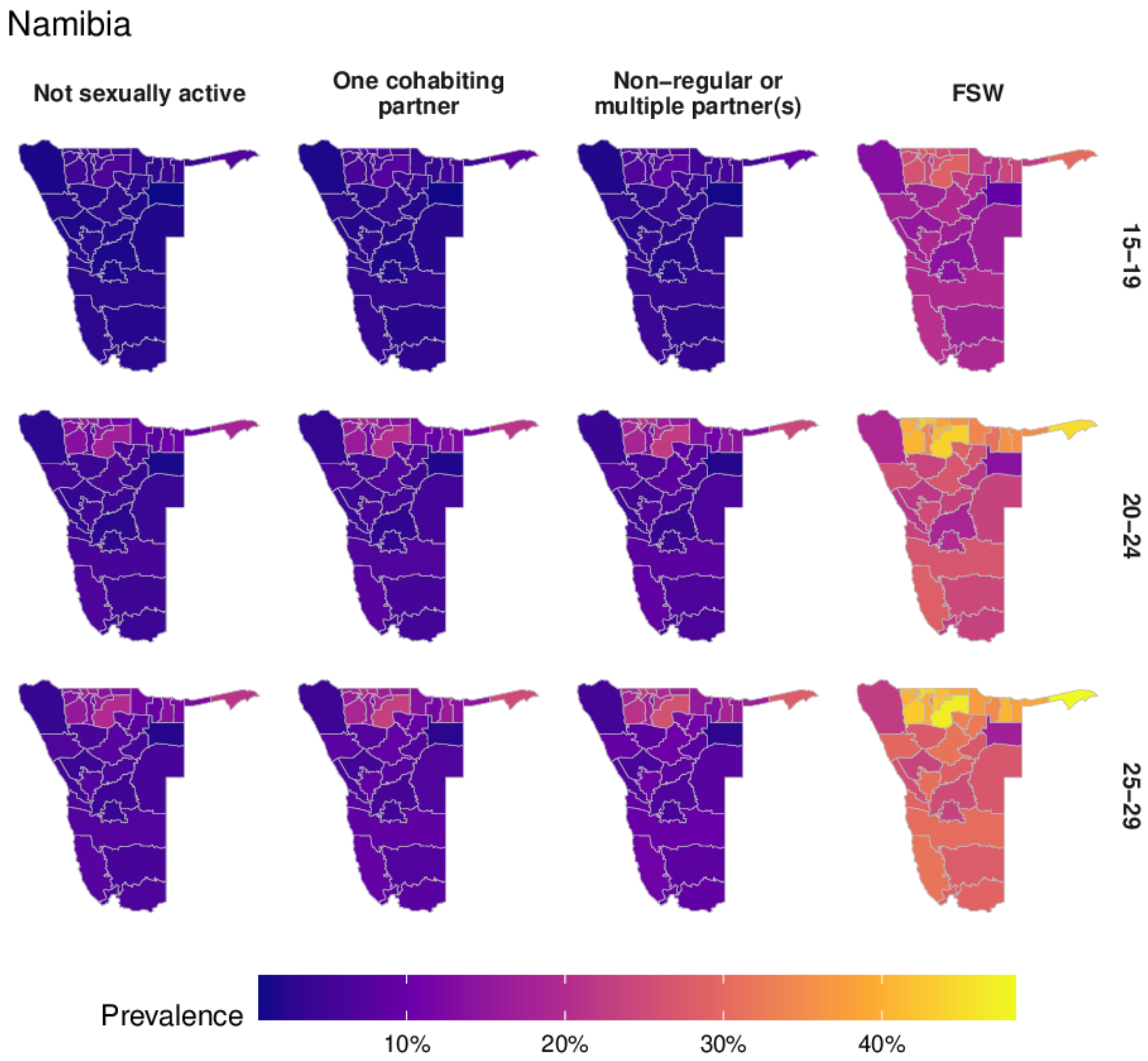

Figure X: District-level HIV prevalence for each of the risk groups in 2018 in Namibia.

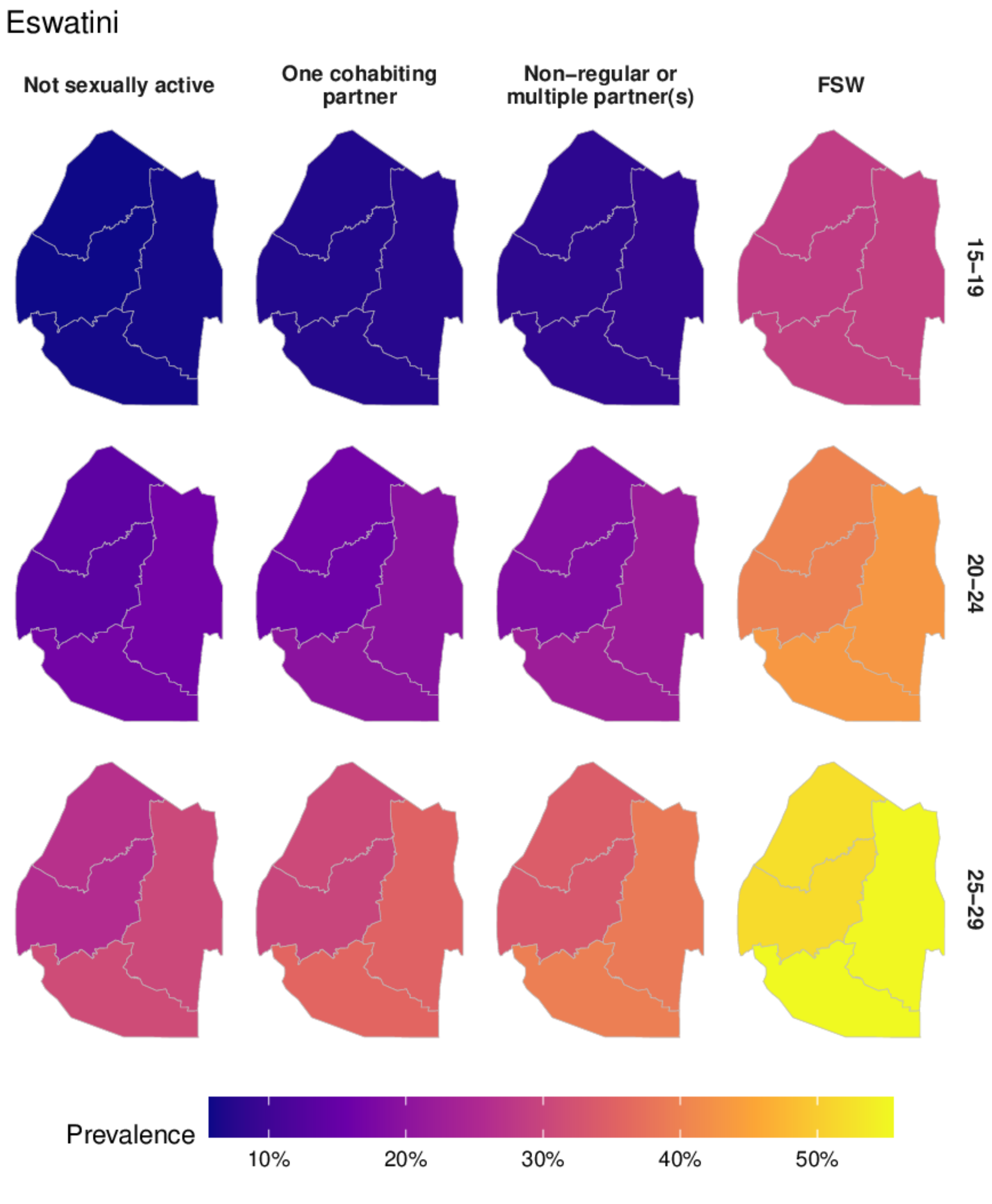

Figure Y: District-level HIV prevalence for each of the risk groups in 2018 in Eswatini.

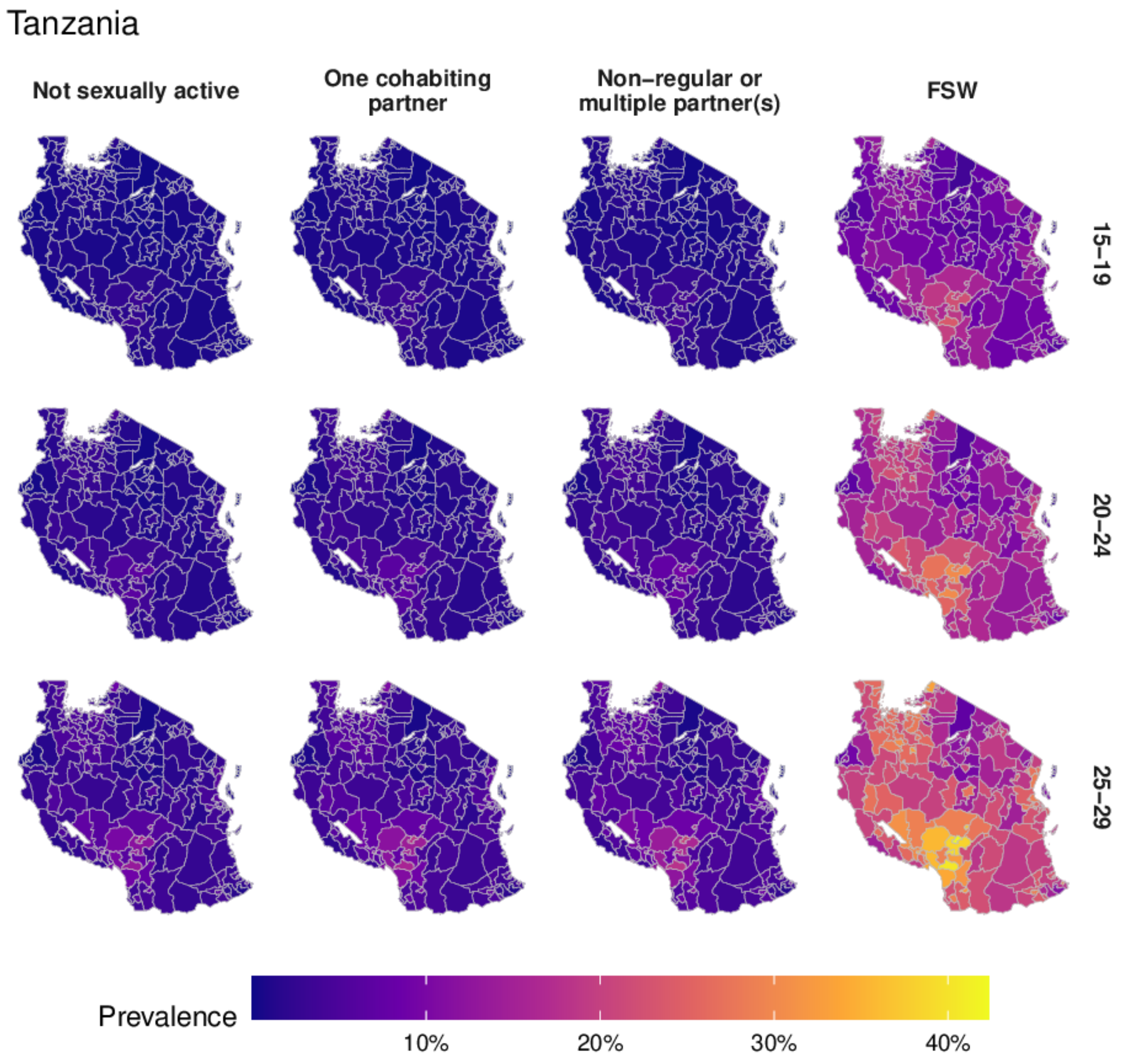

Figure Z: District-level HIV prevalence for each of the risk groups in 2018 in Tanzania.

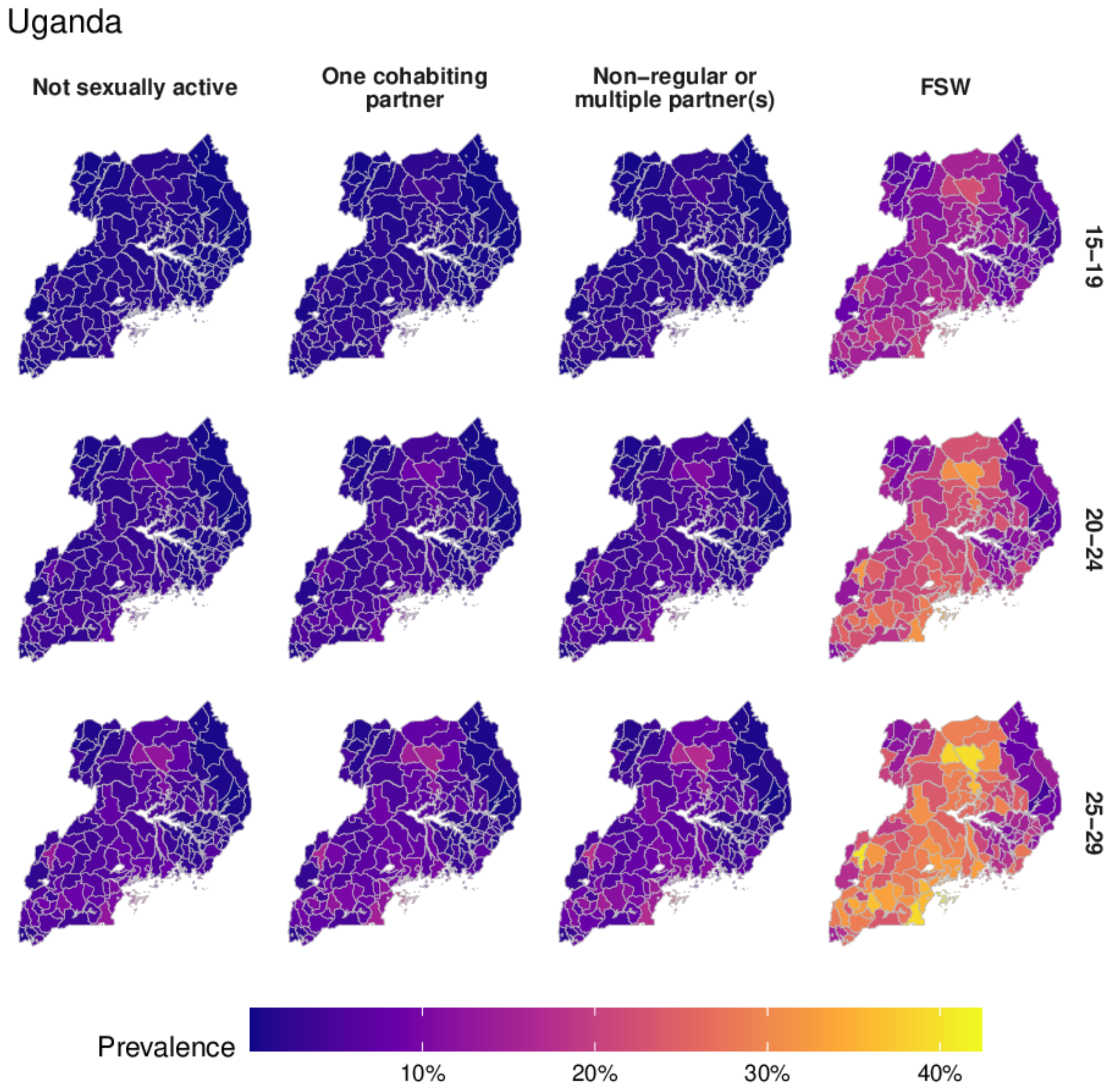

Figure AA: District-level HIV prevalence for each of the risk groups in 2018 in Uganda.

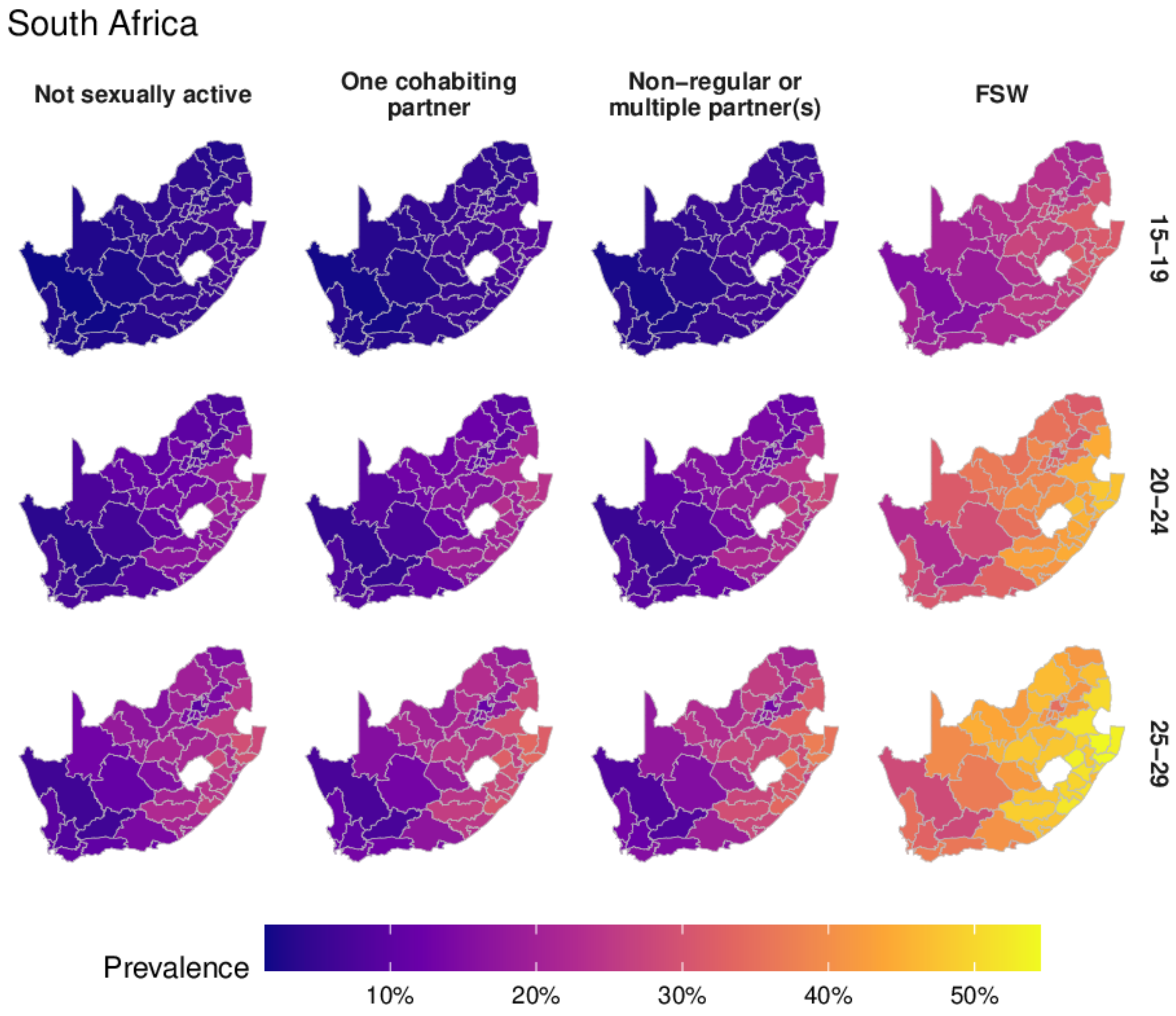

Figure AB: District-level HIV prevalence for each of the risk groups in 2018 in South Africa.

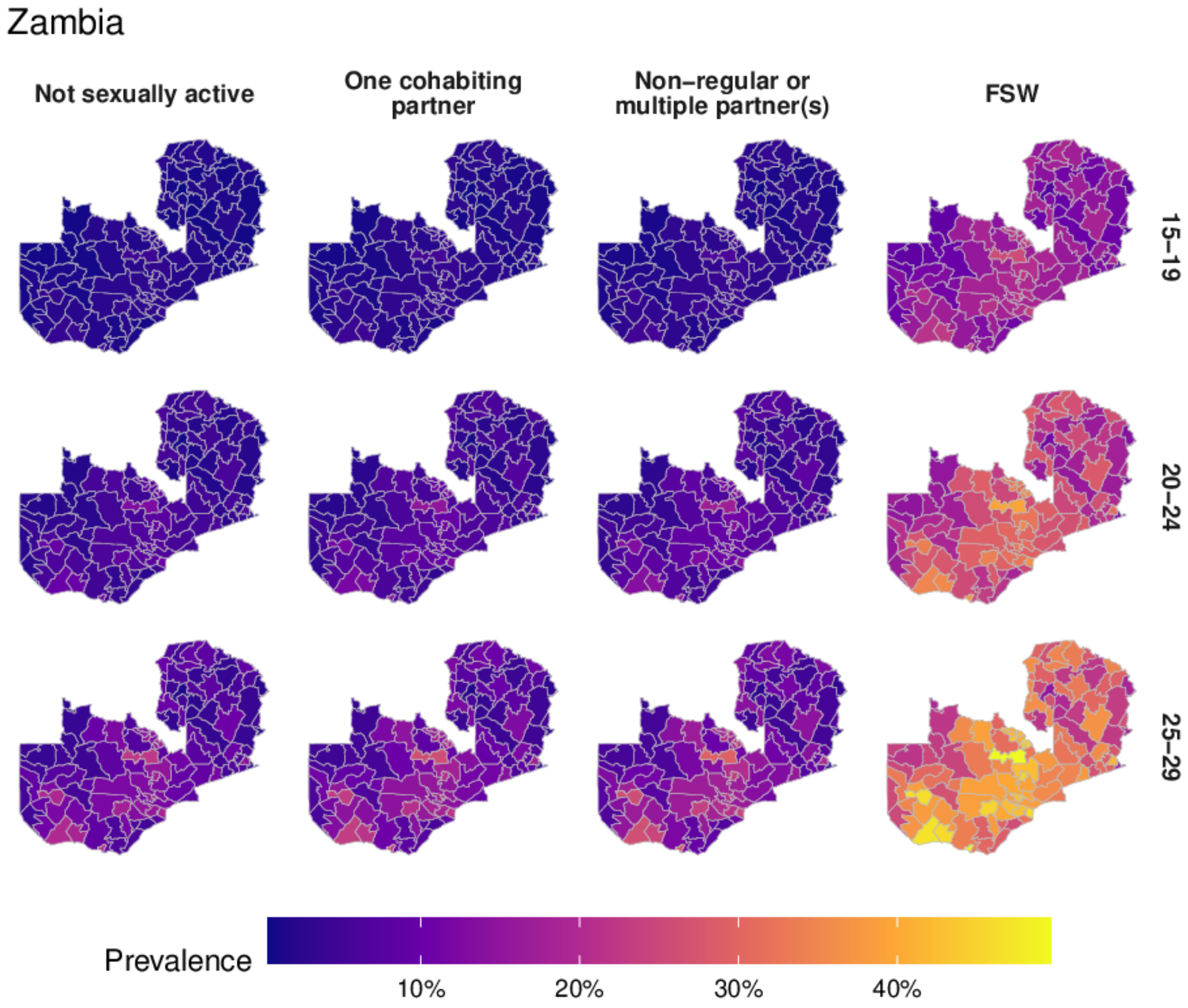

Figure AC: District-level HIV prevalence for each of the risk groups in 2018 in Zambia.

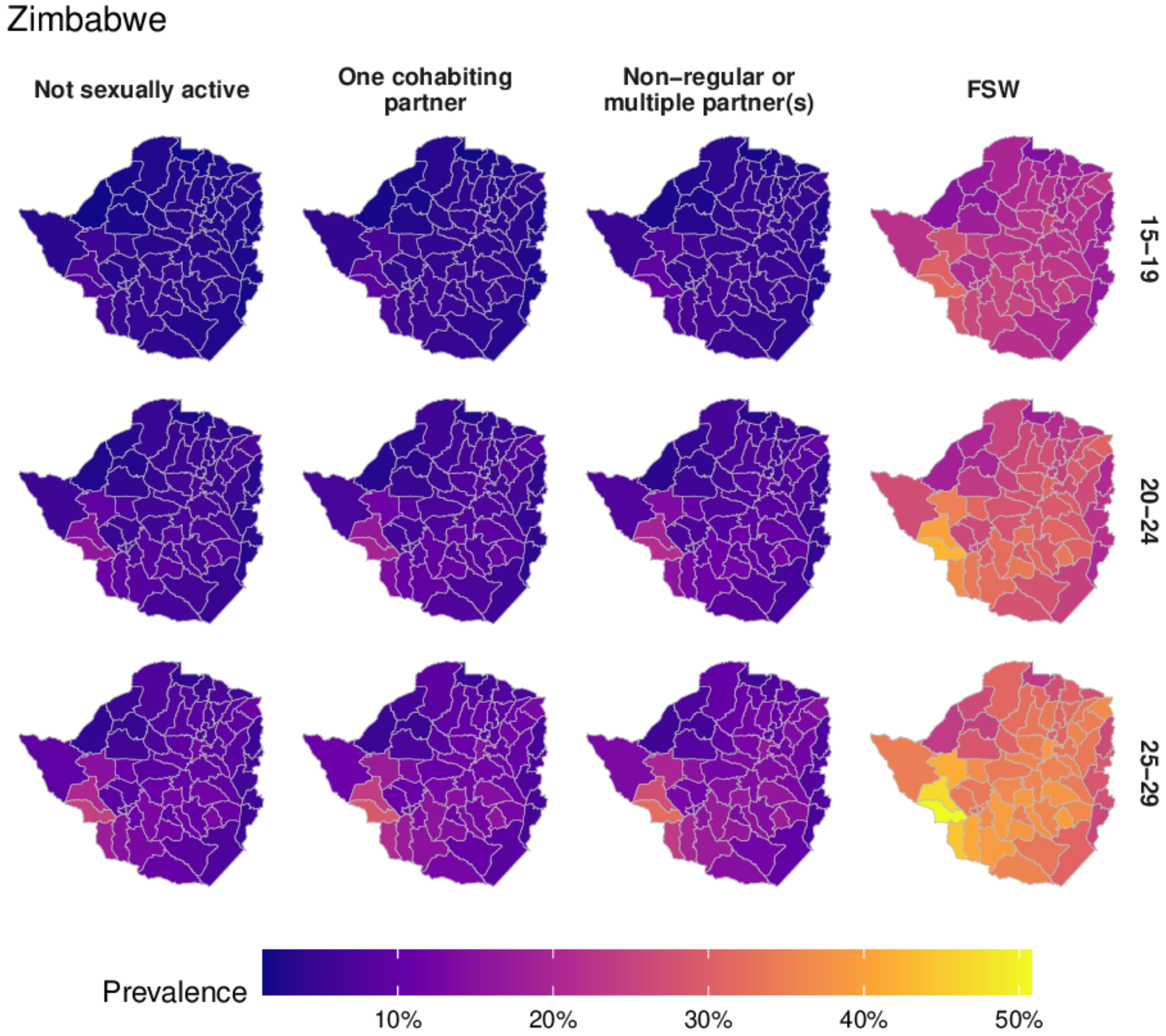

Figure AD: District-level HIV prevalence for each of the risk groups in 2018 in Zimbabwe.

HIV incidence

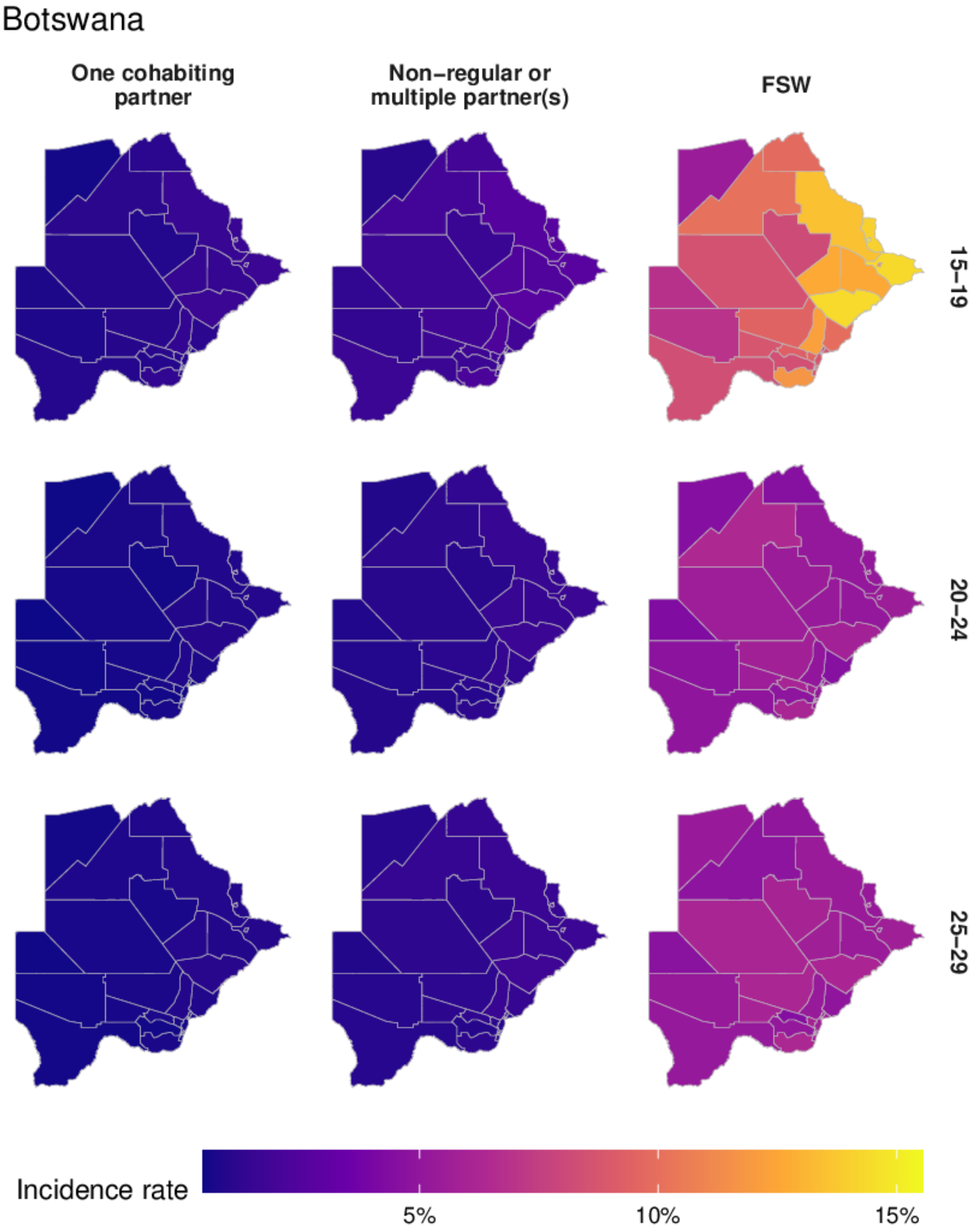

Figure AE: District-level HIV incidence for each of the risk groups in 2018 in Botswana.

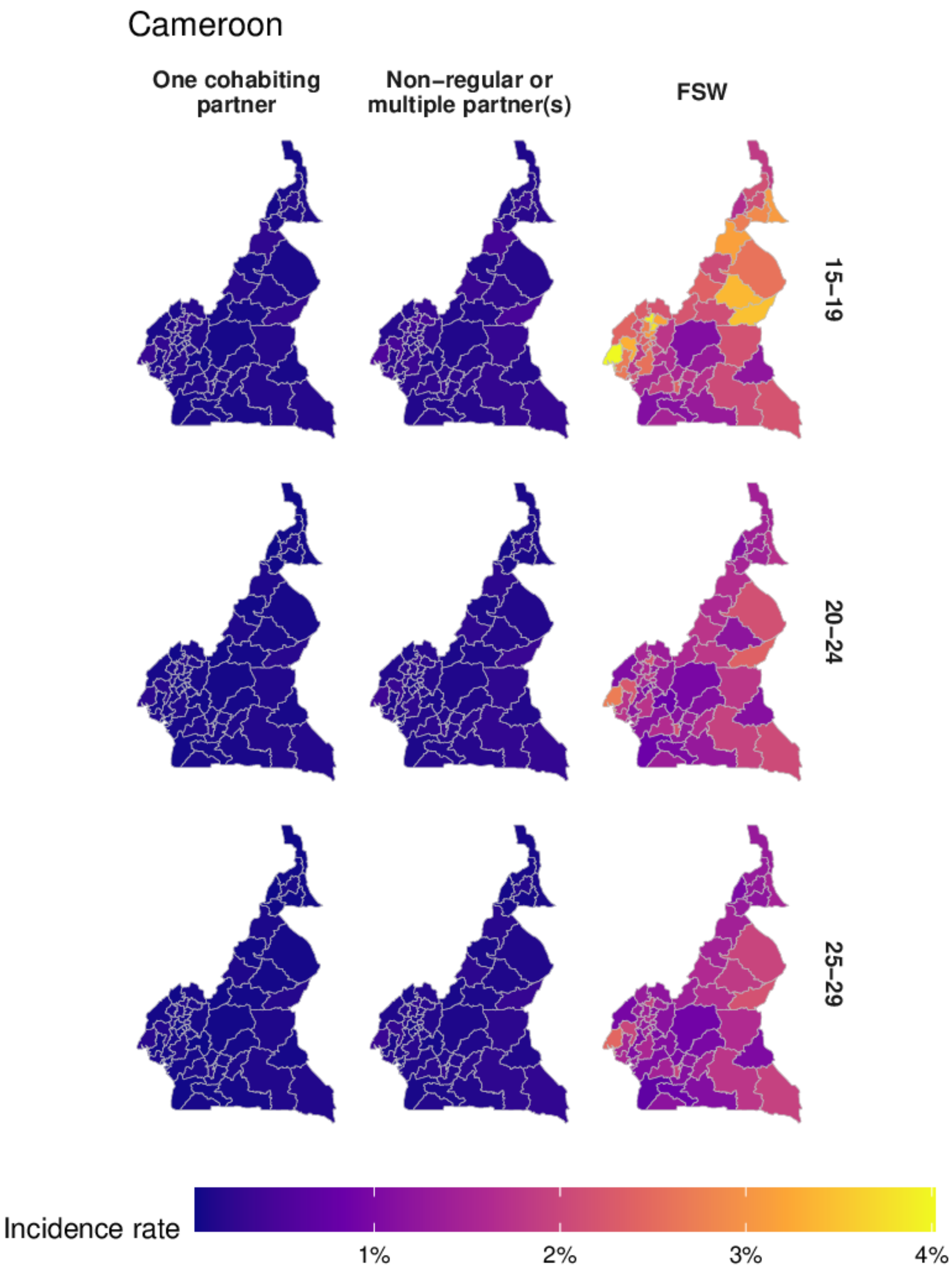

Figure AF: District-level HIV incidence for each of the risk groups in 2018 in Cameroon.

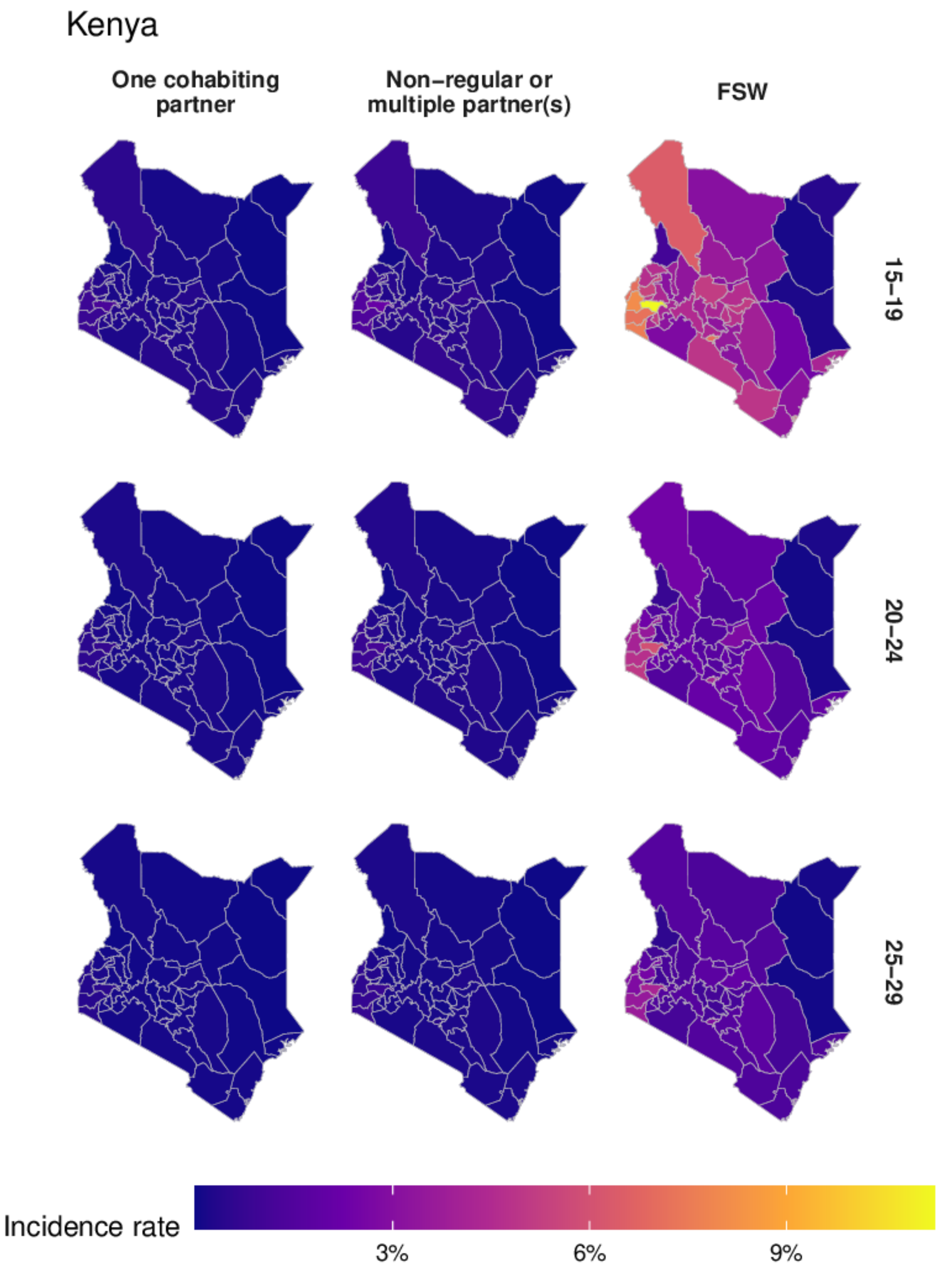

Figure AG: District-level HIV incidence for each of the risk groups in 2018 in Kenya.

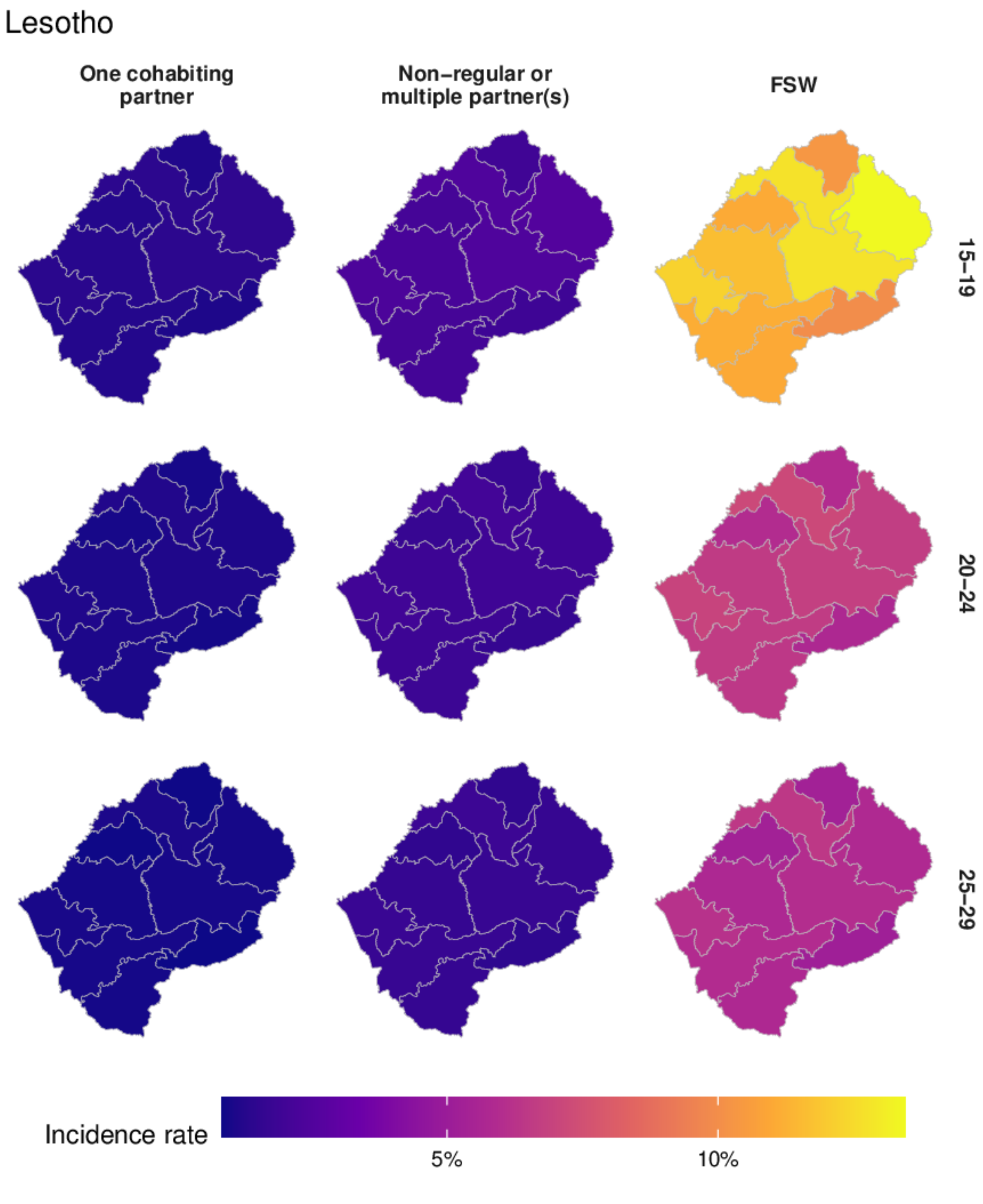

Figure AH: District-level HIV incidence for each of the risk groups in 2018 in Lesotho.

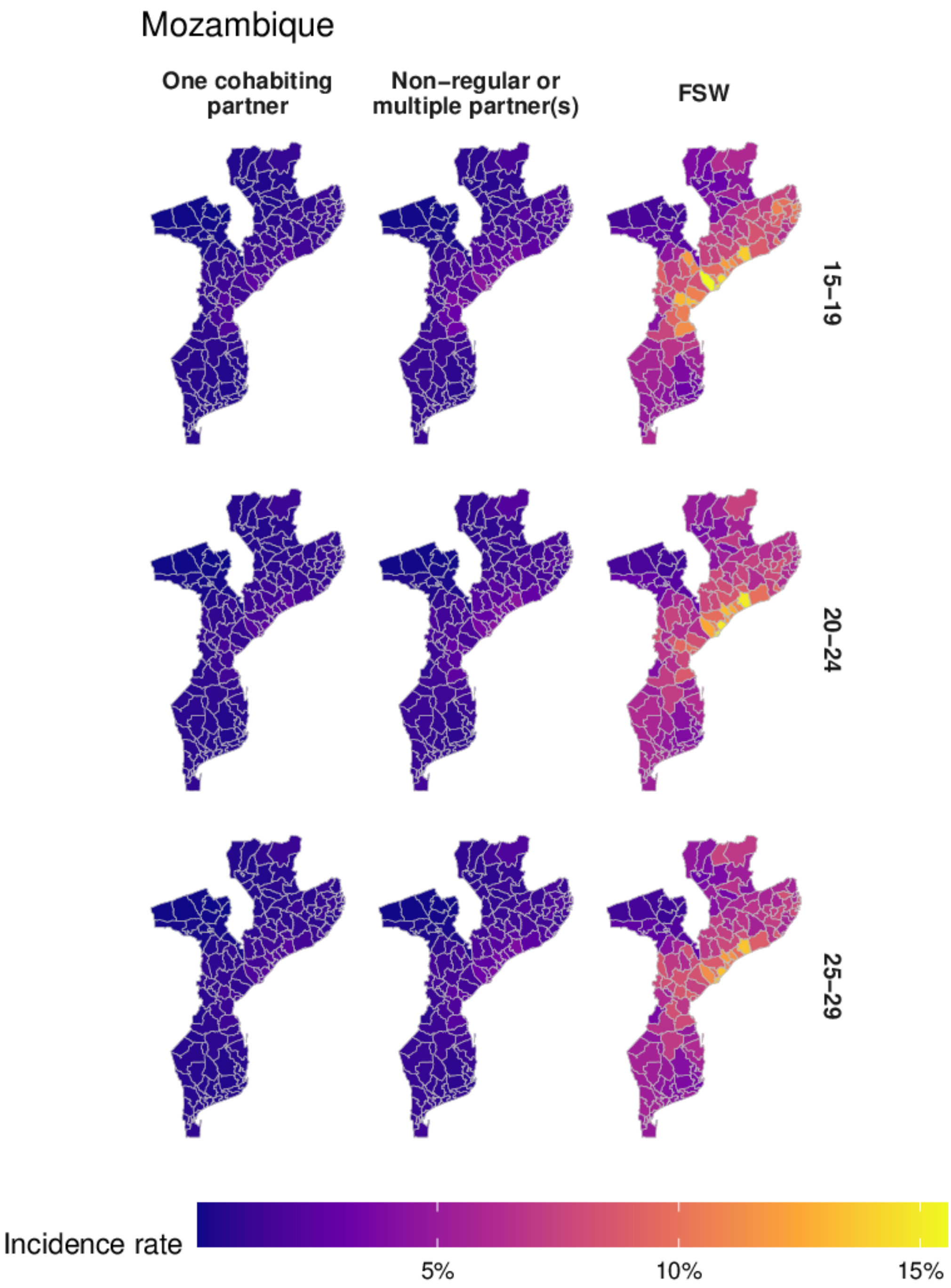

Figure AI: District-level HIV incidence for each of the risk groups in 2018 in Mozambique.

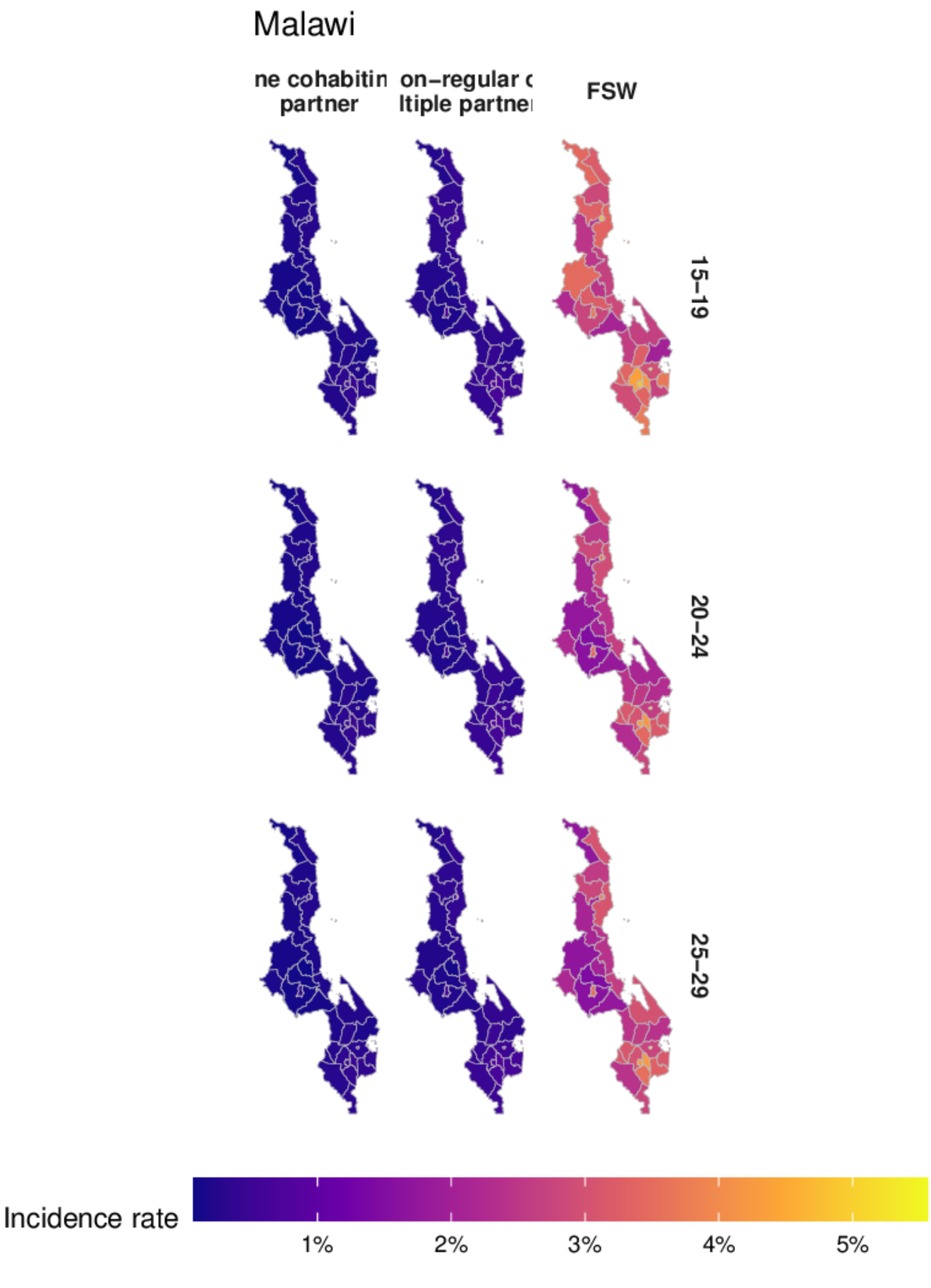

Figure AJ: District-level HIV incidence for each of the risk groups in 2018 in Malawi.

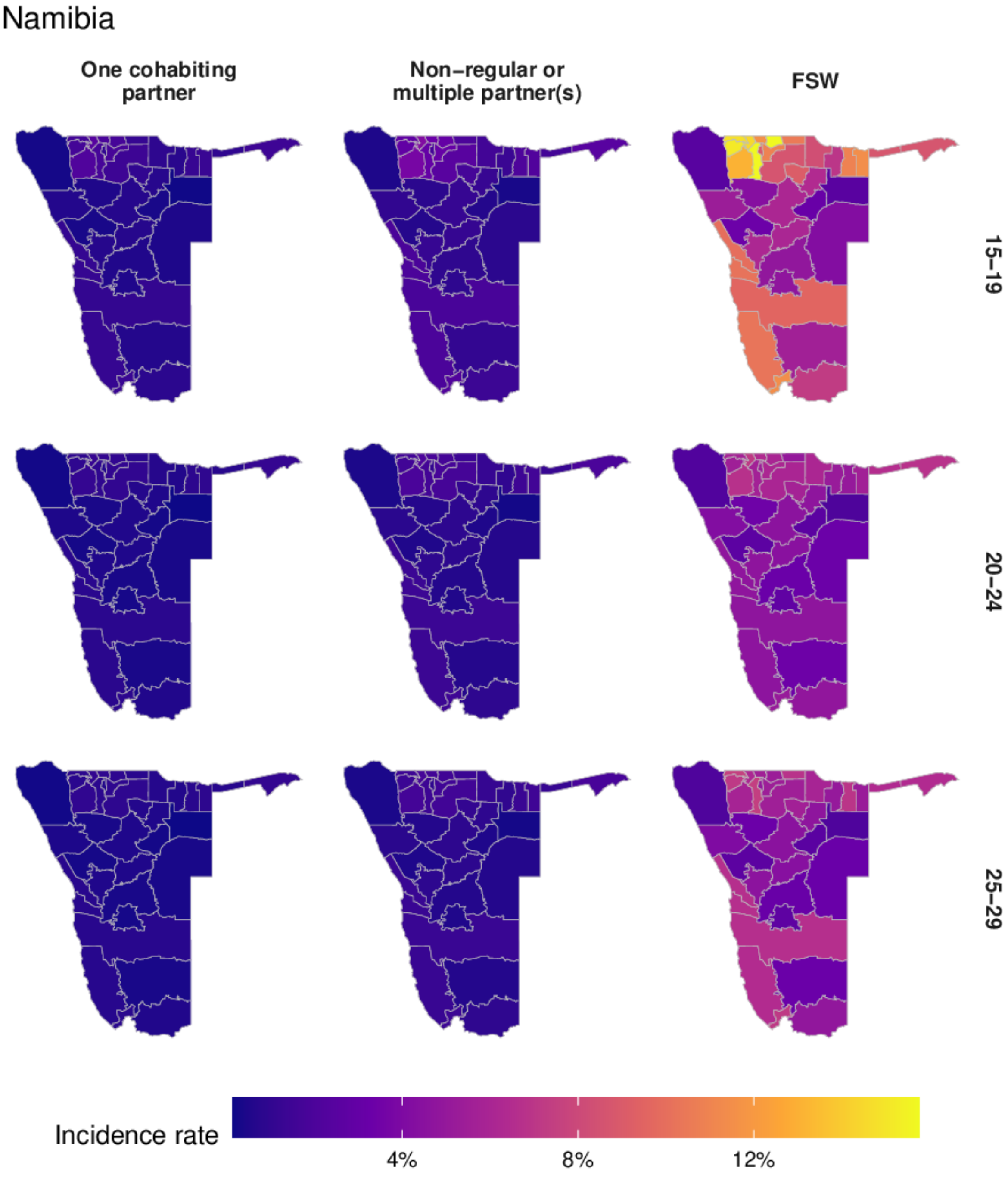

Figure AK: District-level HIV incidence for each of the risk groups in 2018 in Namibia.

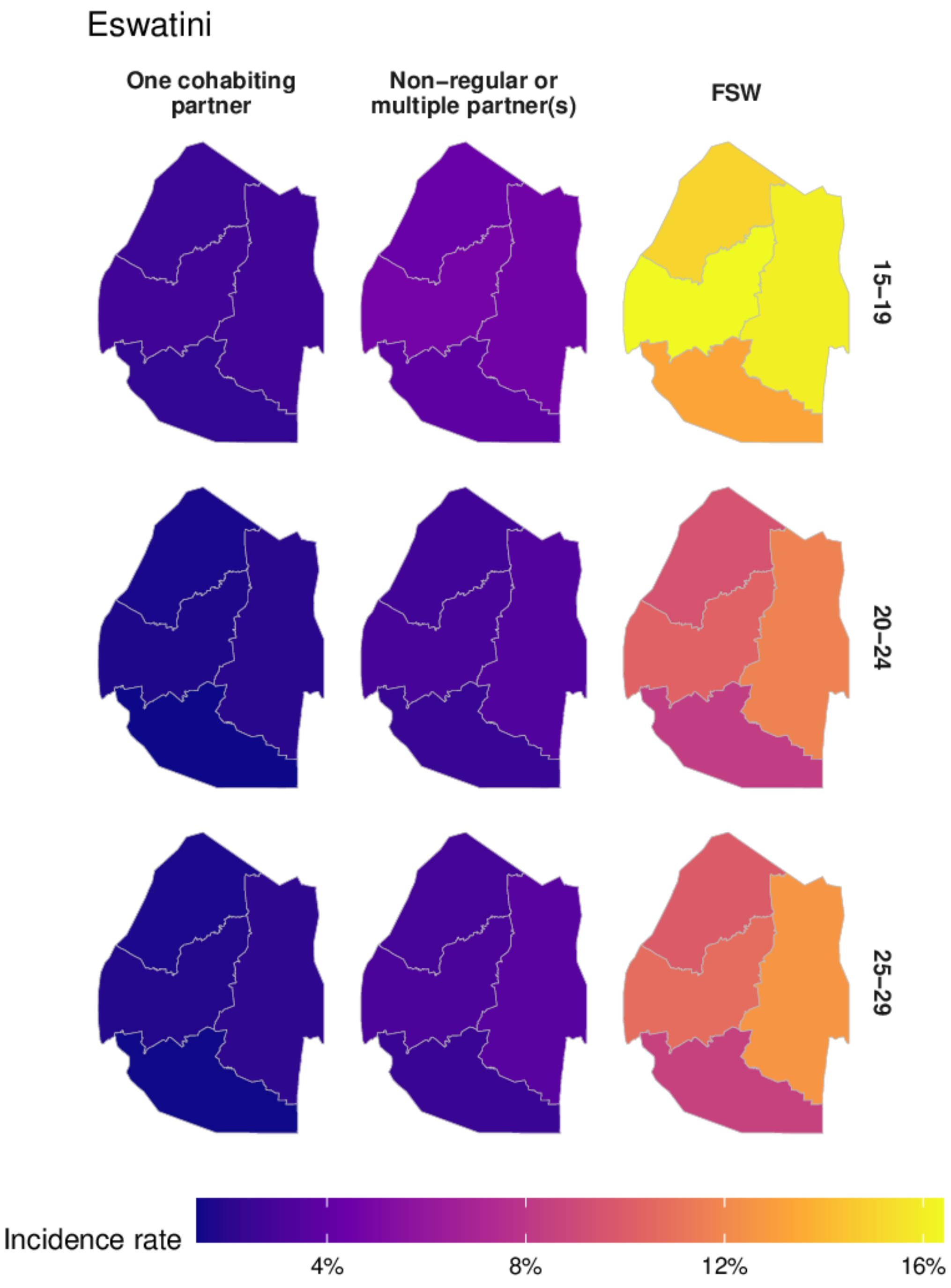

Figure AL: District-level HIV incidence for each of the risk groups in 2018 in Eswatini.

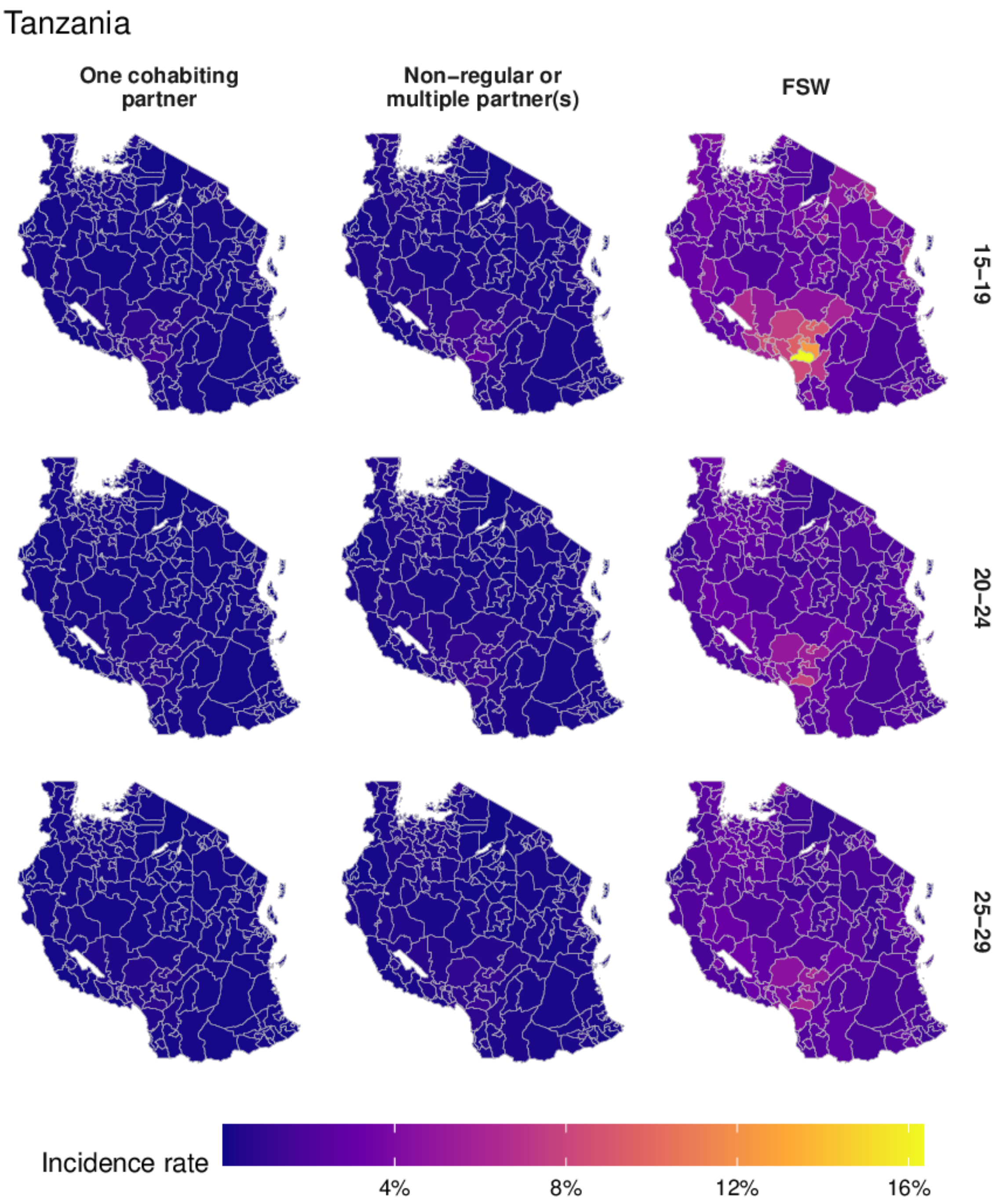

Figure AM: District-level HIV incidence for each of the risk groups in 2018 in Tanzania.

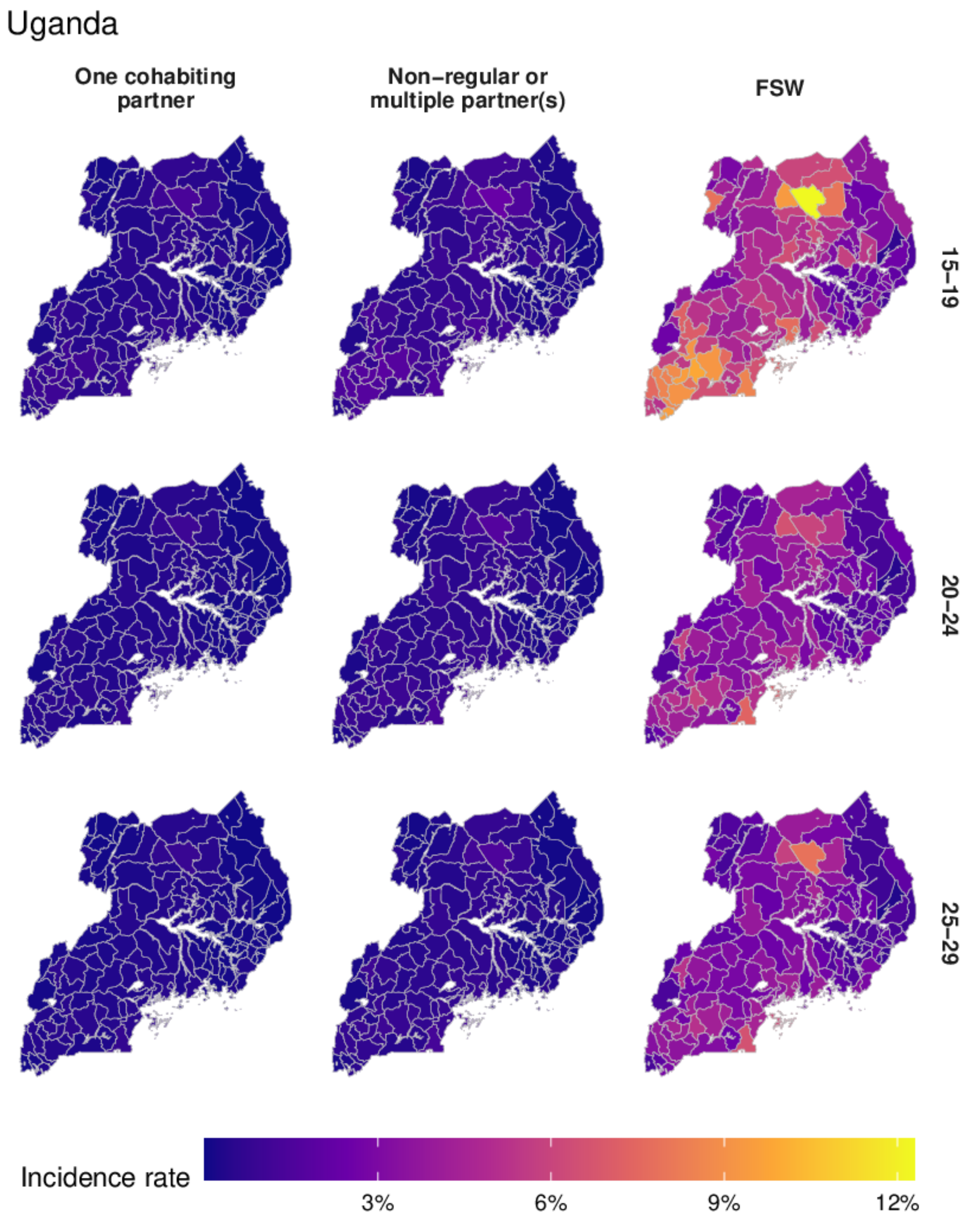

Figure AN: District-level HIV incidence for each of the risk groups in 2018 in Uganda.

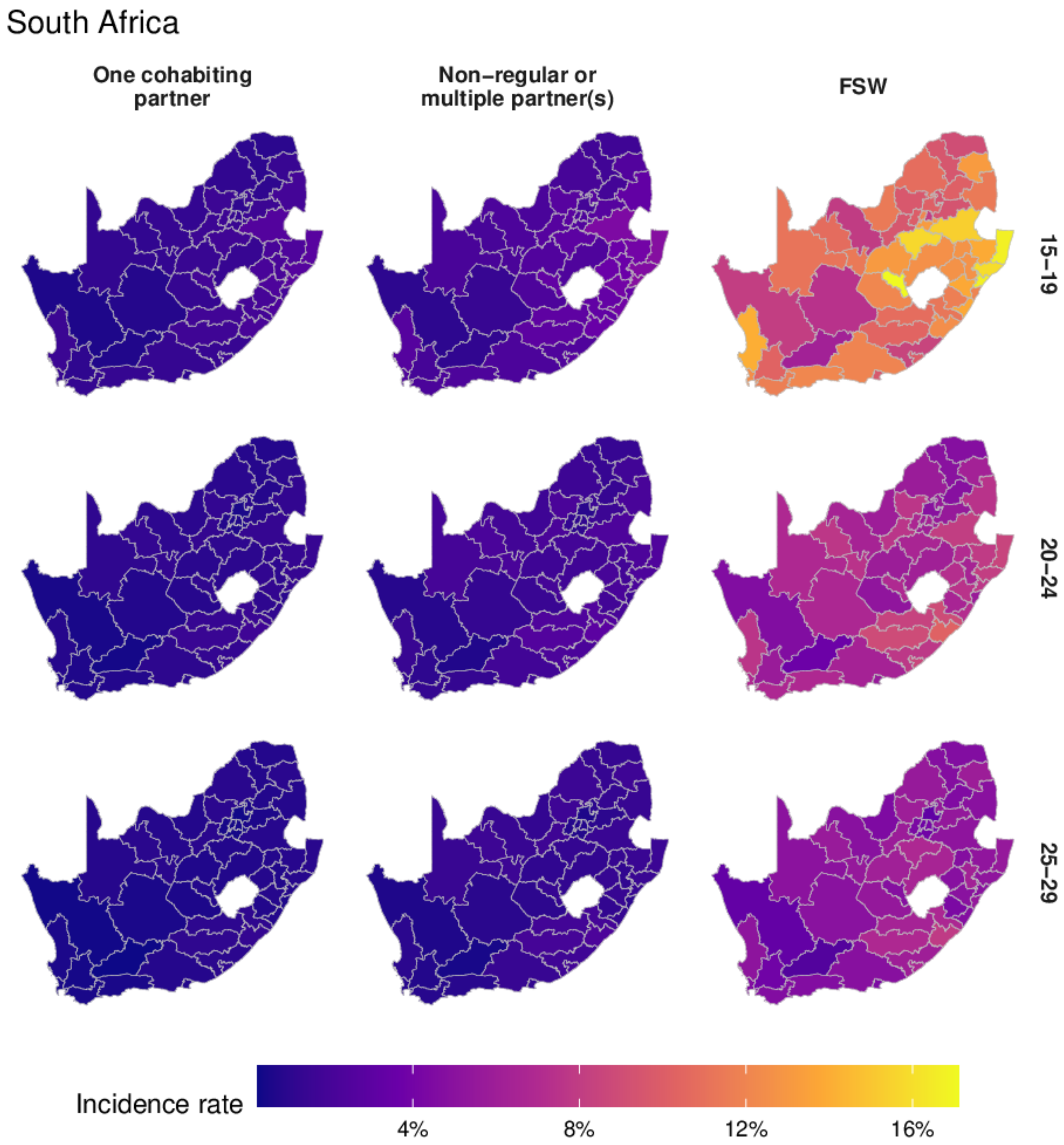

Figure AO: District-level HIV incidence for each of the risk groups in 2018 in South Africa.

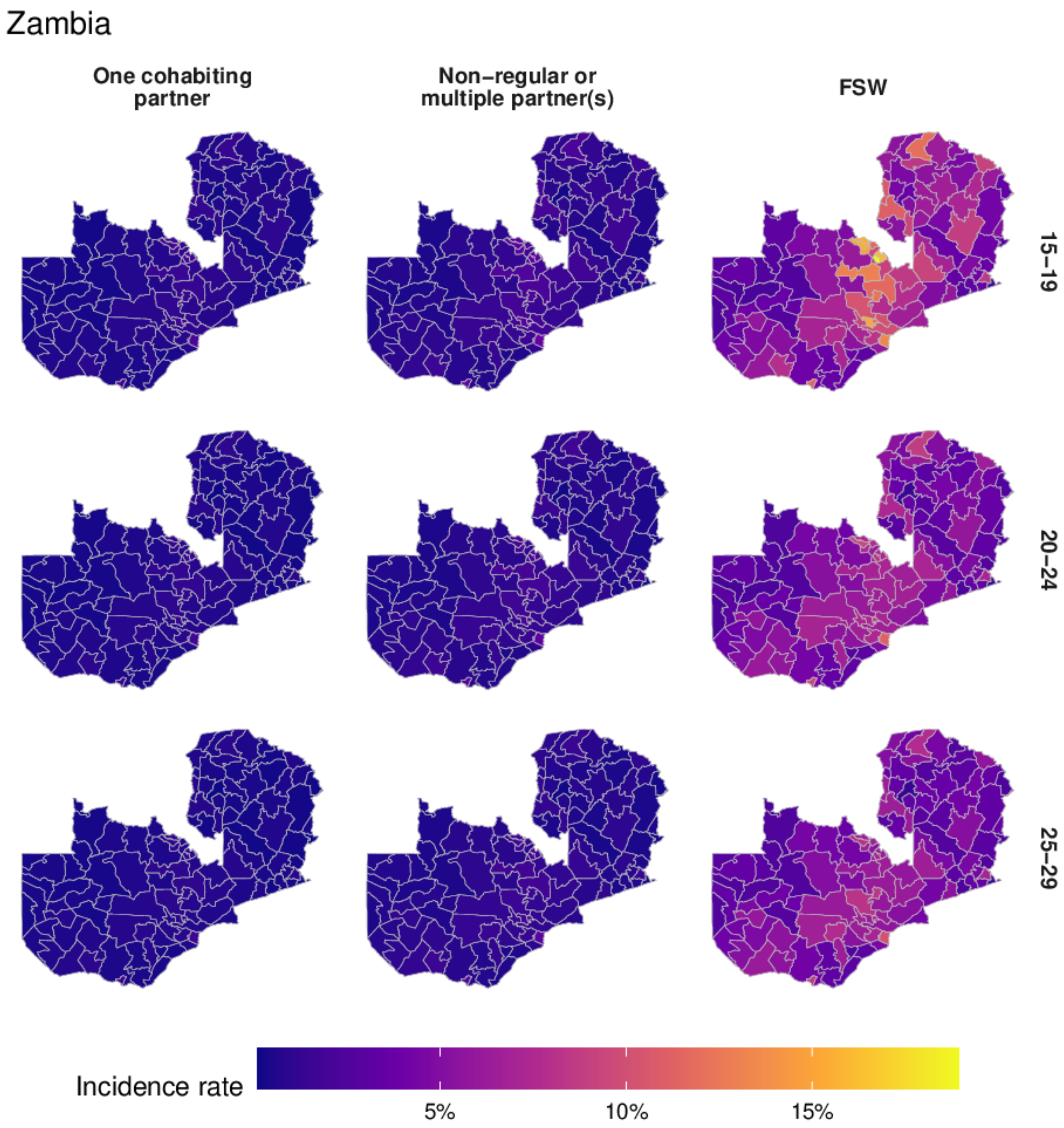

Figure AP: District-level HIV incidence for each of the risk groups in 2018 in Zambia.

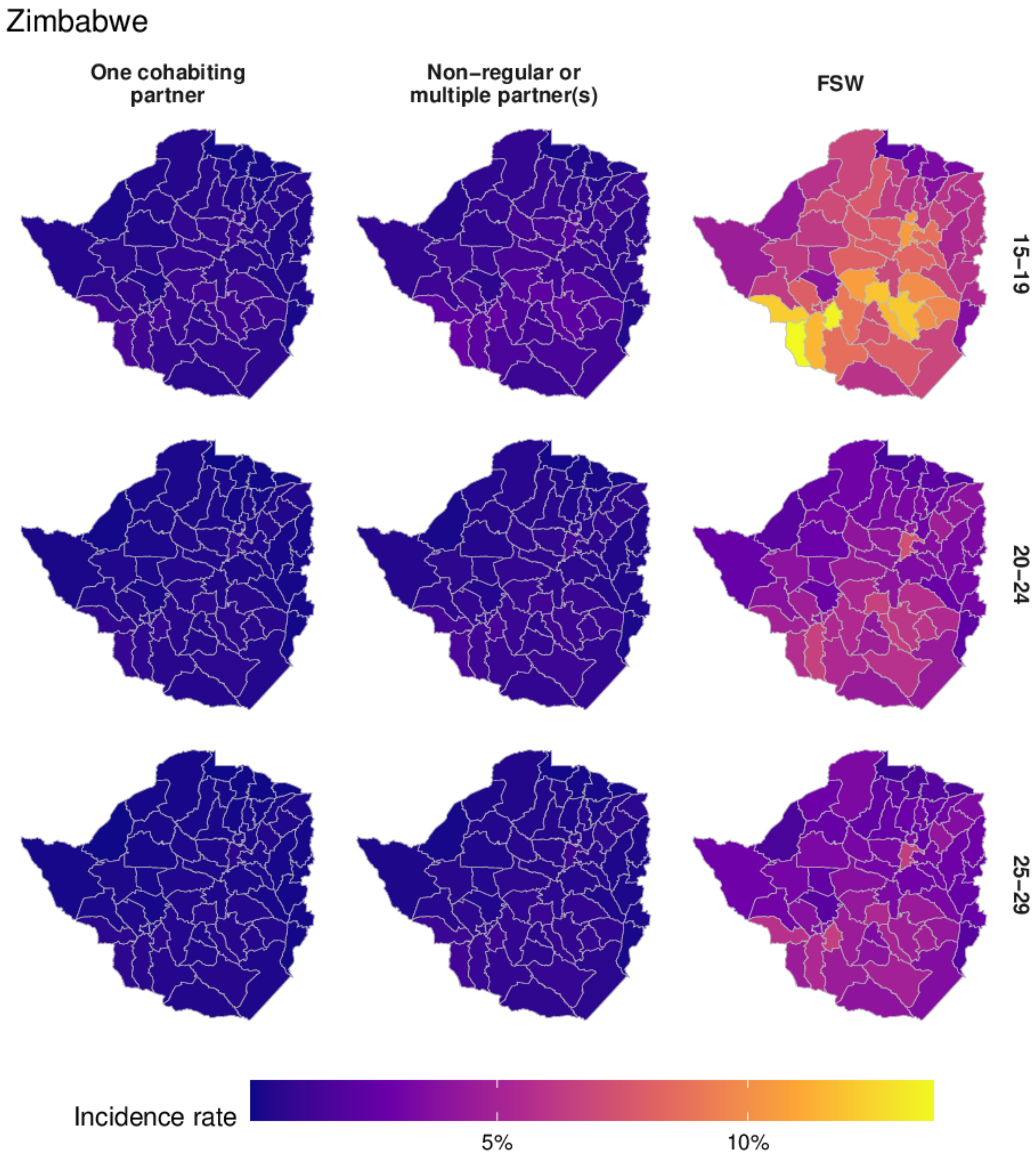

Figure AQ: District-level HIV incidence for each of the risk groups in 2018 in Zimbabwe.

Expected new infections reached

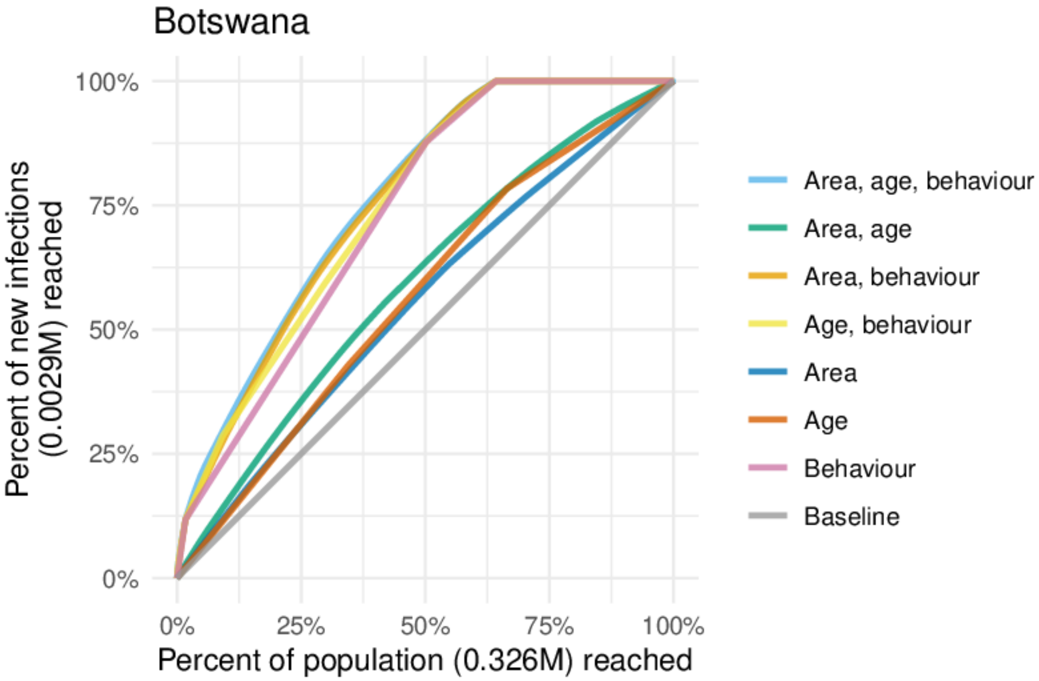

Figure AR: Percentage of expected new infections reached taking a variety of risk stratification approaches against the percentage of at risk population reached in Botswana.

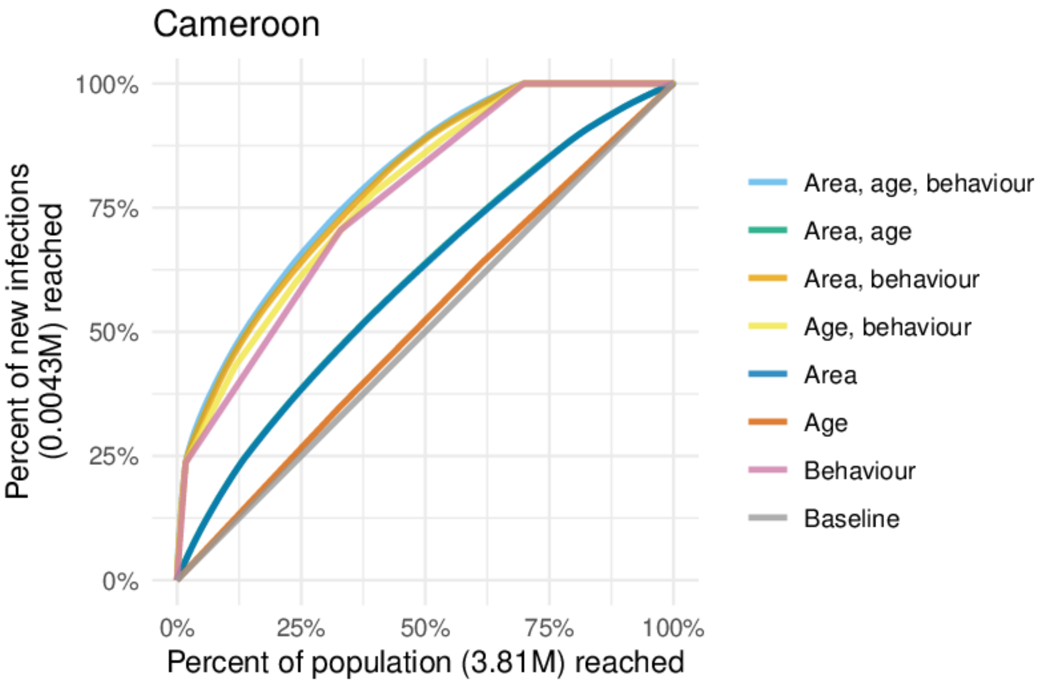

Figure AS: Percentage of expected new infections reached taking a variety of risk stratification approaches against the percentage of at risk population reached in Cameroon.

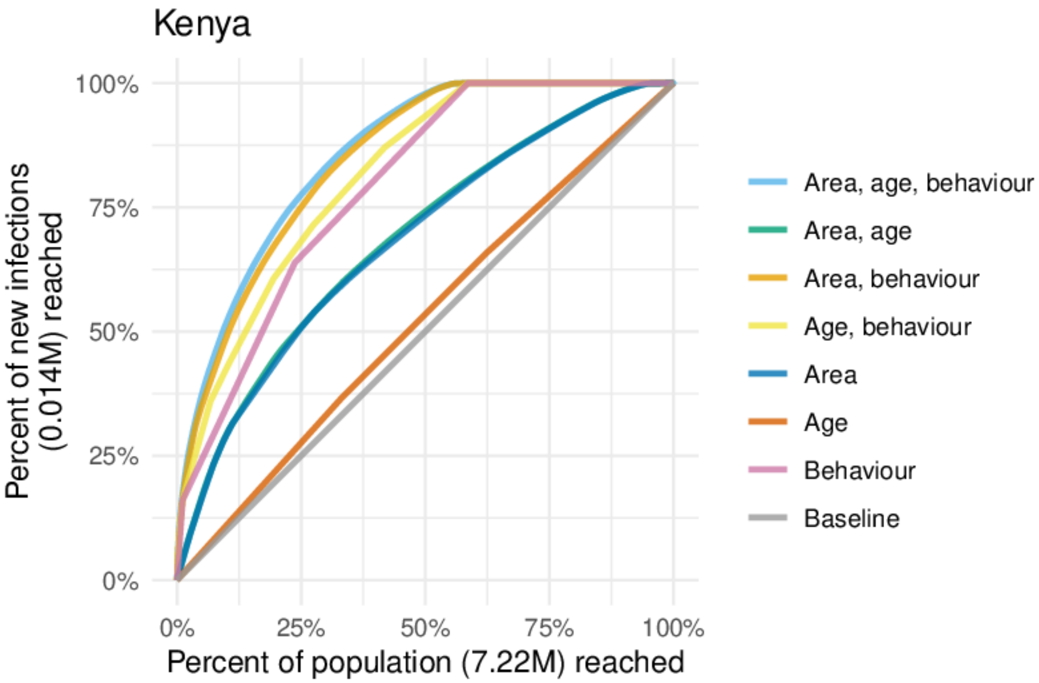

Figure AT: Percentage of expected new infections reached taking a variety of risk stratification approaches against the percentage of at risk population reached in Kenya.

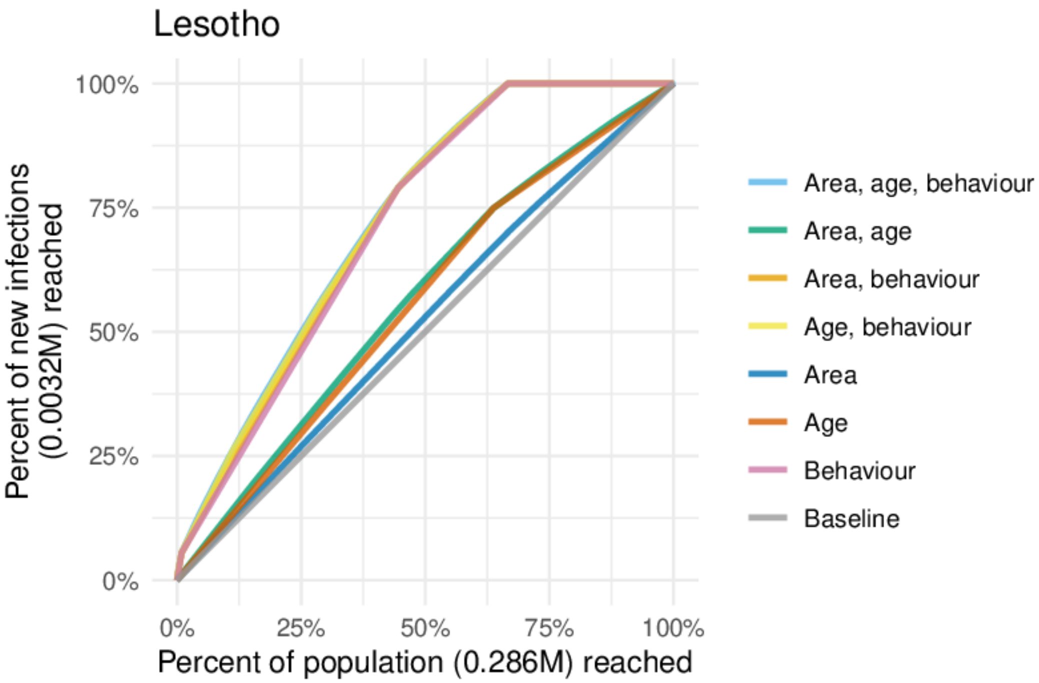

Figure AU: Percentage of expected new infections reached taking a variety of risk stratification approaches against the percentage of at risk population reached in Lesotho.

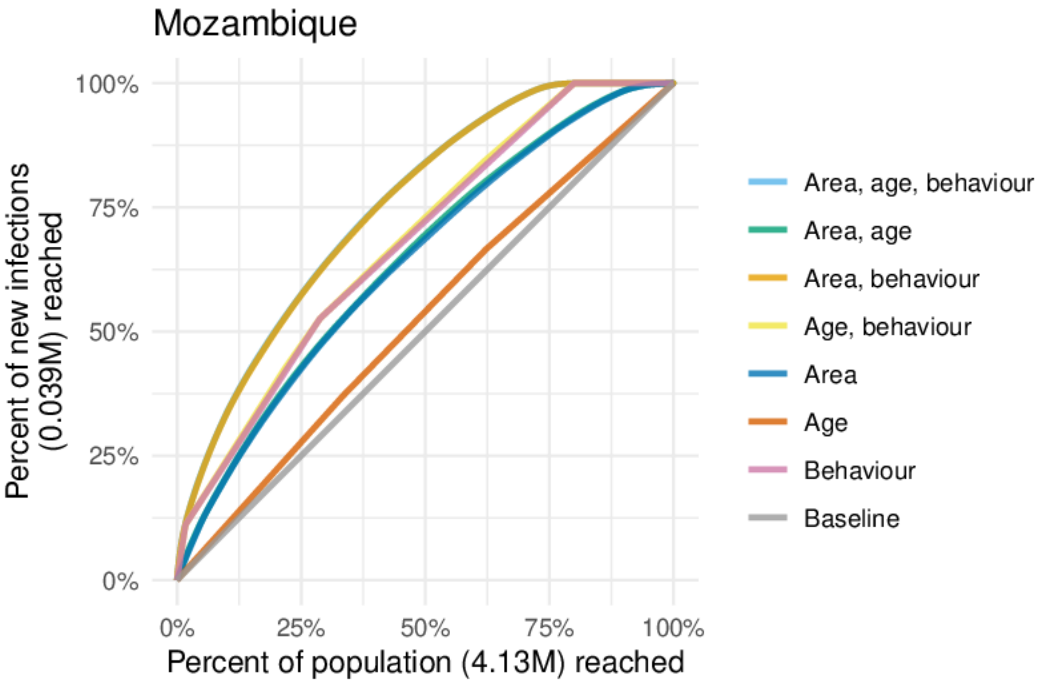

Figure AV: Percentage of expected new infections reached taking a variety of risk stratification approaches against the percentage of at risk population reached in Mozambique.

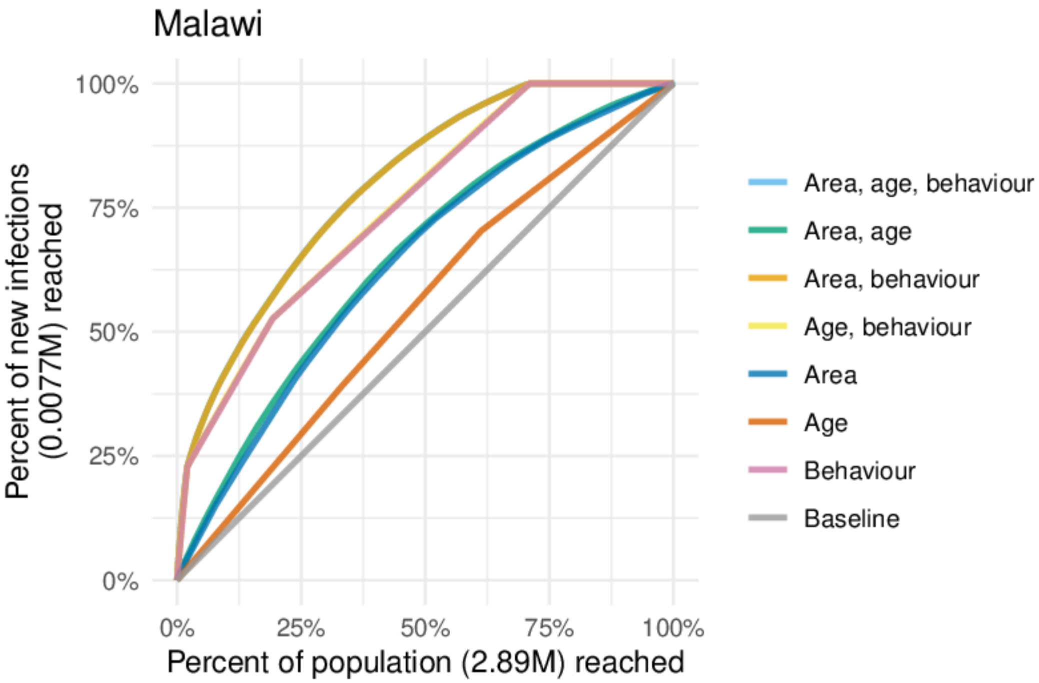

Figure AW: Percentage of expected new infections reached taking a variety of risk stratification approaches against the percentage of at risk population reached in Malawi.

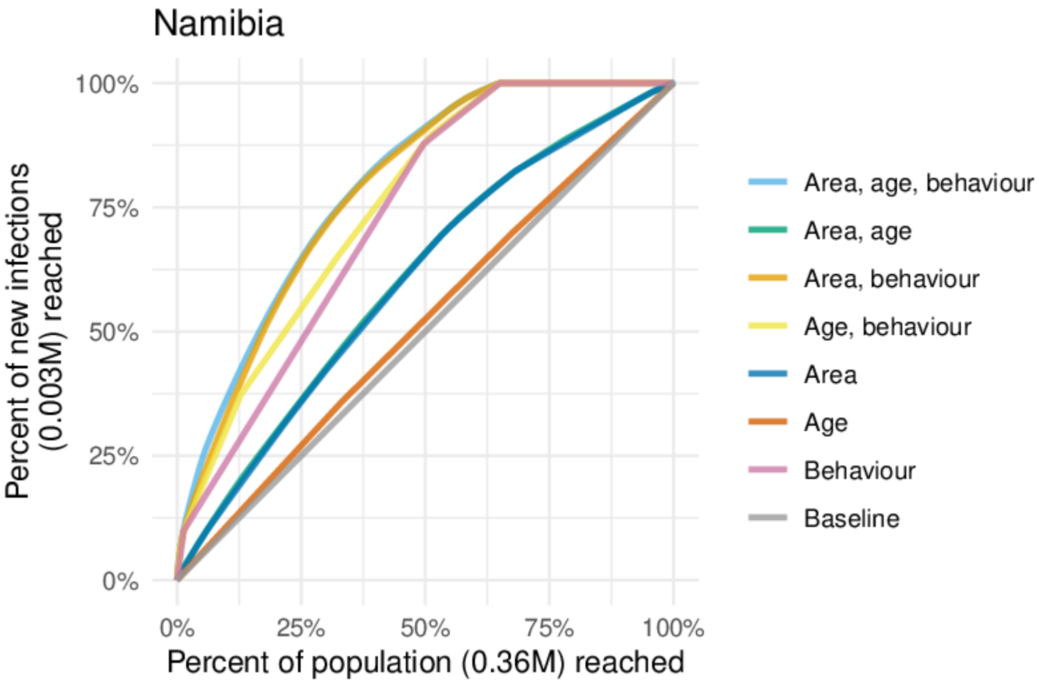

Figure AX: Percentage of expected new infections reached taking a variety of risk stratification approaches against the percentage of at risk population reached in Namibia.

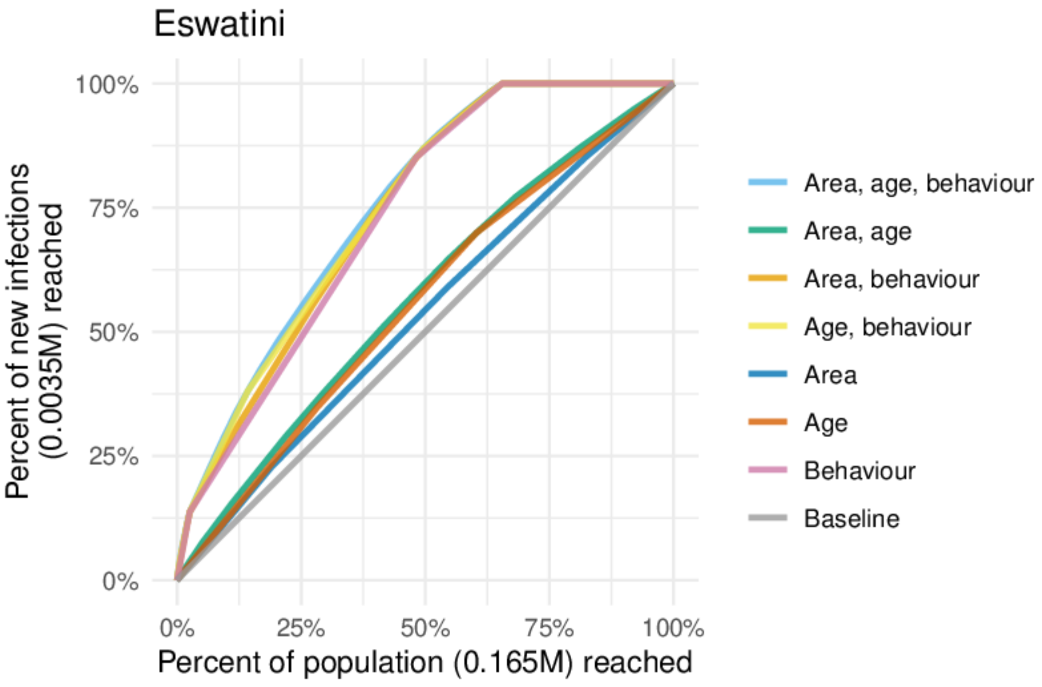

Figure AY: Percentage of expected new infections reached taking a variety of risk stratification approaches against the percentage of at risk population reached in Eswatini.

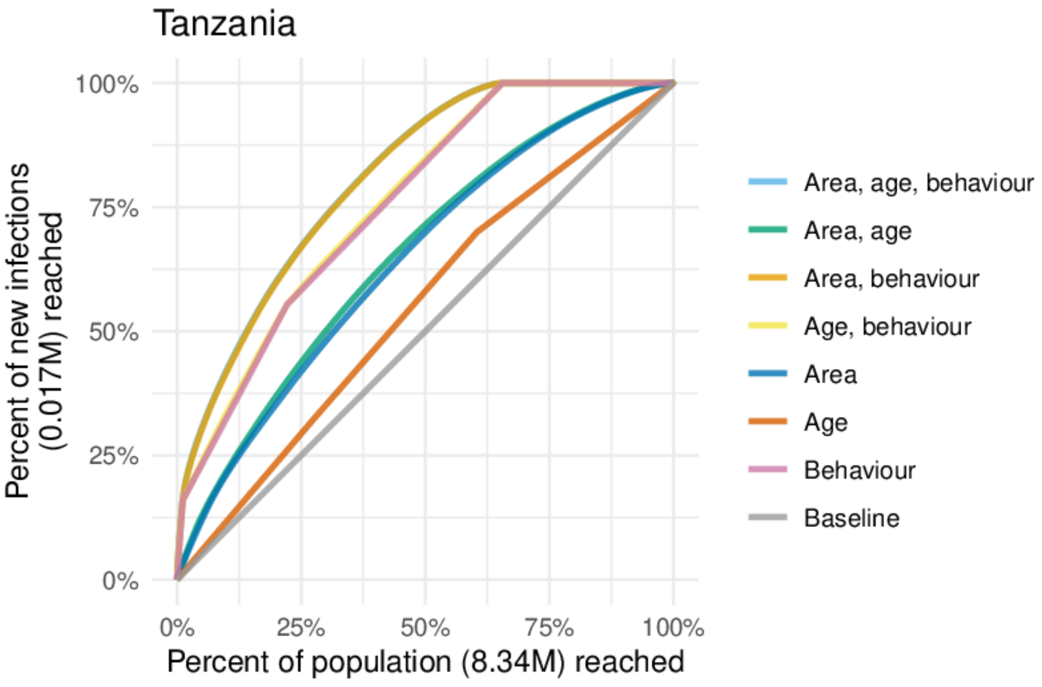

Figure AZ: Percentage of expected new infections reached taking a variety of risk stratification approaches against the percentage of at risk population reached in Tanzania.

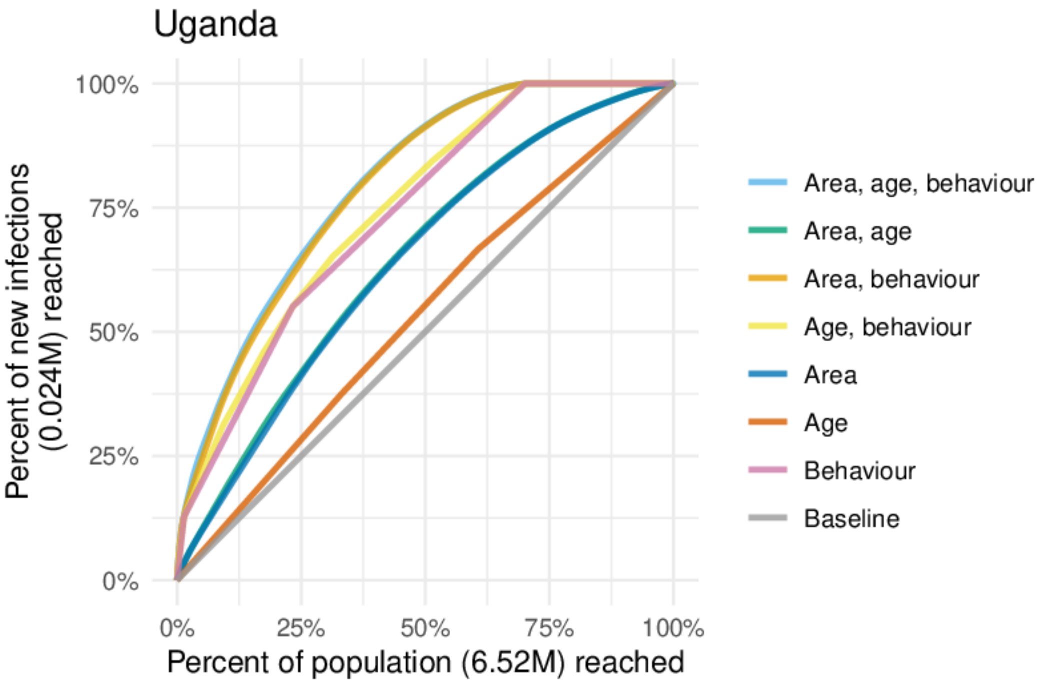

Figure BA: Percentage of expected new infections reached taking a variety of risk stratification approaches against the percentage of at risk population reached in Uganda.

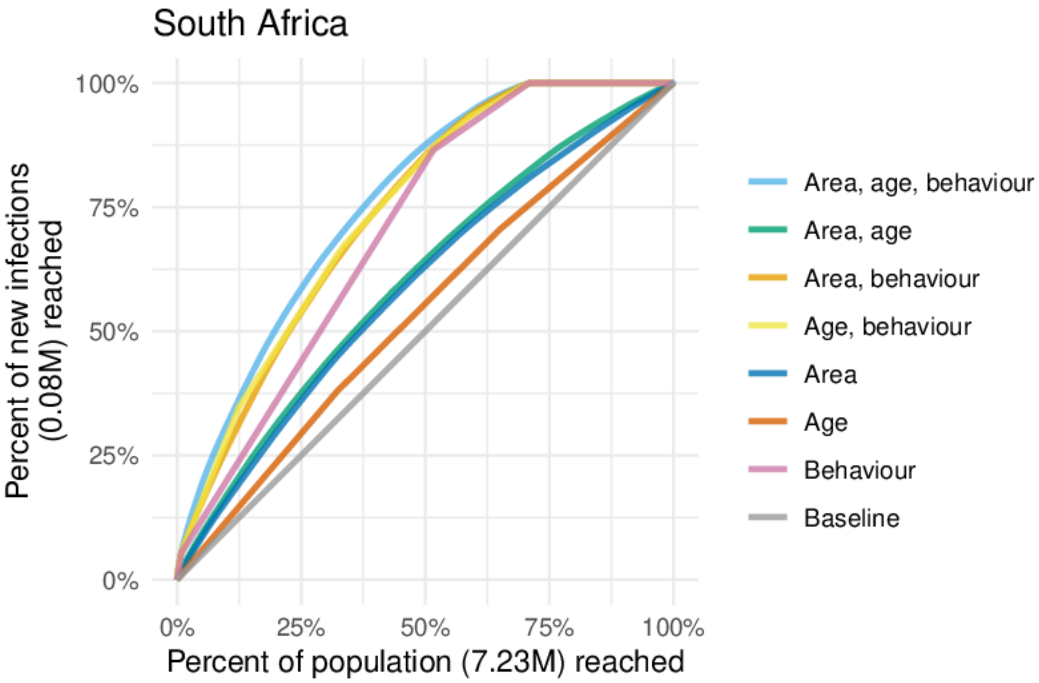

Figure BB: Percentage of expected new infections reached taking a variety of risk stratification approaches against the percentage of at risk population reached in South Africa.

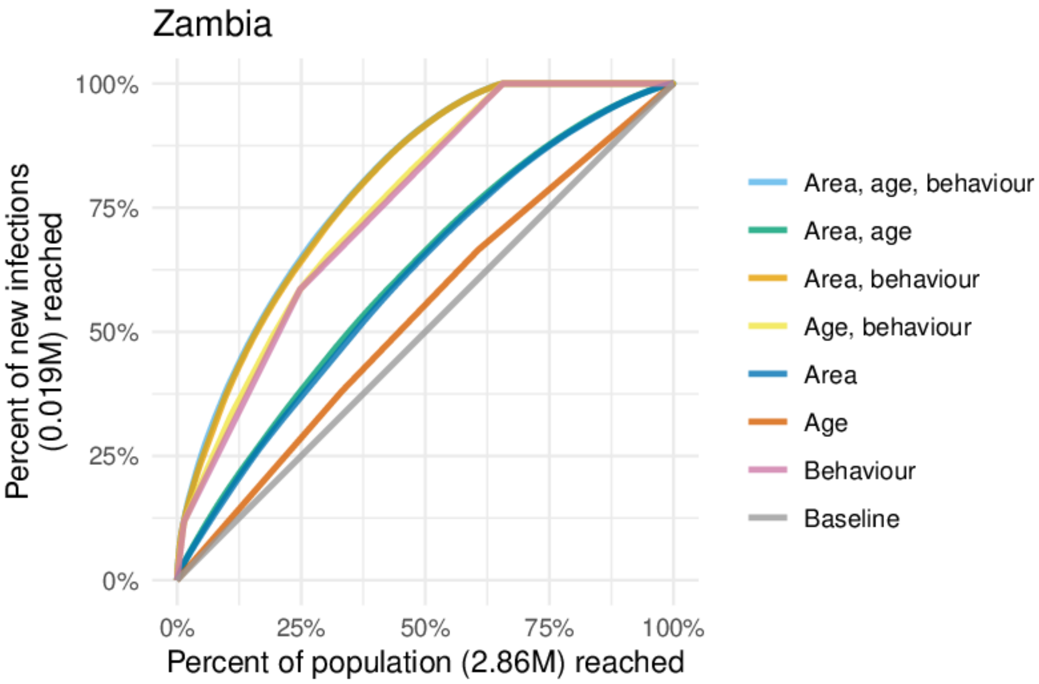

Figure BC: Percentage of expected new infections reached taking a variety of risk stratification approaches against the percentage of at risk population reached in Zambia.

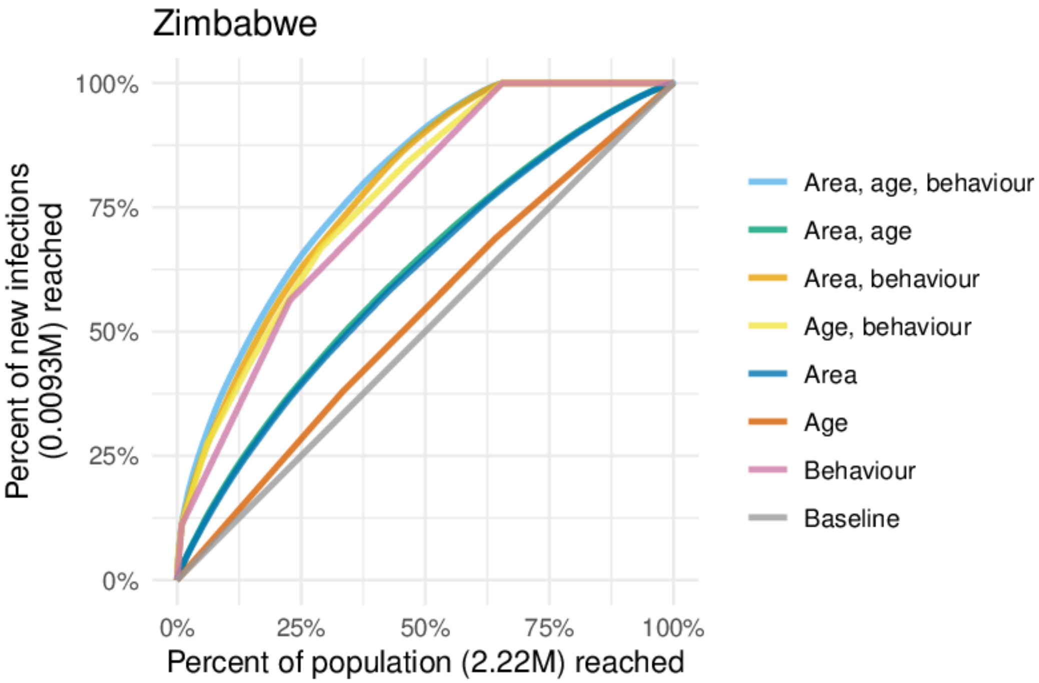

Figure BD: Percentage of expected new infections reached taking a variety of risk stratification approaches against the percentage of at risk population reached in Zimbabwe.
