# Supplementary material for: Spatio-temporal estimates of HIV risk group proportions for adolescent girls and young women across 13 priority countries in sub-Saharan Africa
Source: PLOS Glob Public Health. 2023 Apr 19;3(4):e0001731. doi: 10.1371/journal.pgph.0001731 (PMC10115274; doi:10.1371/journal.pgph.0001731)
Supplement: Attachment — Submitted filename: review.pdf [file pgph.0001731.s004.pdf]

Response to reviewers and editorial comments for "Spatio-temporal estimates of HIV risk group proportions for adolescent girls and young women across 13 priority countries in sub-Saharan Africa"

Corresponding author: Adam Howes (ath19@ic.ac.uk)

Contents

Reviewer 1 **2**

Reviewer 2 **4**

Editorial comments **5**

References **7**

Reviewer 1

We thank the reviewer for their helpful comments regarding the statistical modelling.

> *1. In the manuscript, explain why you use the INLA not WINBUGS coding? The multinomial regression could be modeled in WINBUGS directly.*

The reviewer is right to note that multinomial logistic regression models can be implemented in probabilistic programming languages like `WinBUGS` directly. However, for this application, Markov chain Monte Carlo approaches would be prohibitively expensive. For this reason, we chose to use integrated nested Laplace approximations via `R-INLA`, which have been shown to have comparable accuracy for latent Gaussian models in the realistic, pre-asymptotic regime. We have added the following text to the methods section of the manuscript (lines 126-129) to clarify this point:

> For models with a Gaussian latent field, INLA has comparable accuracy to Markov chain Monte Carlo with realistic, finite samples, and is substantially more computationally tractable for high dimensional models like ours, which has 940 districts, 20 years, 3 age groups, and 4 risk groups.

> *2. Is there no other potential covariate that could be used for better modeling?*

Many of the covariates which one might expect to be most predictive of risk group proportions are themselves difficult to accurately measure, and can only lead to modest at best improvements in model performance. For example, despite the case for their being a clear link between the "proportion clients of FSW" covariate and the "proportion FSW" outcome we found only marginal benefits to inclusion.

We agree that identifying predictive and measurable predictors of risk group proportions, or high risk locations, is an important area for further research. We have commented on this in the discussion section (lines 316-321):

> More generally, we did not consider covariates potentially predictive of risk group proportions (such as sociodemographic characteristics, education, local economic activity, cultural and religious norms and attitudes), which are typically difficult to measure spatially. Identifying measurable correlates of risk, or particular settings in which time-concentrated HIV risk occurs, is an important area for further research to improve risk prioritisation and precision HIV programme delivery.

> *3. Please explain sub-national effect more clearly. Why and how you used it?*

We did not use the specific term "sub-national effect" in the manuscript. We believe the reviewer is referring to the term "spatial effect", which we use to describe district-specific effects that allow the model to capture district-level variation in risk group proportions, primarily informed by survey data in each district.

Thank you to the reviewer for highlighting the omission of specific definition for this term. Throughout the manuscript, we have replaced references to "effect" by the more accurate "random effect", and have clarified the usage of the term spatial random effect where it first appears in the methods section (lines 108-114) as follows:

> All models included intercepts for each risk group, as well as age, country, and age-country random effects. To account for district-level variation we used spatial random effects consisting of a parameter for each district. We considered alternative model specifications in which the spatial random effects were either independent or spatially correlated such that more information was shared between neighboring districts than those far apart. Similarly, we used temporal random effects to allow variation in risk group proportions over time, and considered alternative model specifications as independent versus first-order auto-regressive where a smooth temporal trend is assumed.

> *4. Why the interaction term for spatiotemporal effect didn't consider in the modeling framework?*

We share the reviewer's interest in the spatiotemporal interaction in risk group proportions, which would allow variation in temporal trend by country. However, we found that these interactions made the model computationally intractable to estimate given the large number of districts, years, age groups and risk groups involved. This is a limitation of our analysis, but, in practice, we do not believe this has a large impact on our results because overall we found that risk group proportions were very stable over time, while they

varied substantially spatially. More specifically, when we fit the model to each country individually, which can be considered as an extreme version of country-specific spatiotemporal interactions, the proportion of variance (Sobol' index) attributable to the temporal (survey) random effects[1] was on average 2.5800954/% (Supplementary Figure B.3). This is corroborated by the lack of temporal trends in Supplementary Figures B.5 through B.17 which show the modelled and direct estimates for each country individually, as well as the fact that unstructured (IID) rather than structured (AR1) temporal random effects were preferred in the model selection (Supplementary Figure A.1).

[1]For those countries where such a random effect could be meaningfully defined.

Reviewer 2

This is a well-crafted manuscript investigating the spatio-temporal estimates of HIV risk group proportions for adolescent girls and young women across 13 priority countries in sub-Saharan Africa. Their analyses identify specific age groups at the district level that should be targeted for HIV intervention in SSA. This is critical in reducing the HIV epidemic in the southern region of SSA. In addition to the main figures, the supplementary Tables and Figures show country-by-country risk, mostly among female sex workers for all age groups. With the help of their models, specific resources can target at-risk populations with a moderate assurance of how many people to reach and where these resources should go.

We thank the reviewer for their kind comments.

My little concern is about using different data from UNAIDS Key Population Atlas apart from the DHS, which is the may source data for the analyses. I believe the two variants of data are based on different designs, and combining them may not result in dependable results. It would have been more attainable if the UNAIDS data had been used in their sensitivity analysis to confirm the results from the DHS data.

For the FSW risk group, we used age-disaggregated (Supplementary Figure A.4) national-level estimates from Stevens et al. (2022) to inform the national risk group population size within each age group, and household survey data to inform subnational variation. Estimates of hidden populations like FSW from household surveys have significant limitations due in part to stigma around disclosing membership, as well as potential for not being included in the sampling frame (Abdul-Quader, Baughman, and Hladik 2014). For this reason, we believe it is more appropriate to calibrate our estimates to Stevens et al. (2022), who as well as including the KP Atlas data, integrate data from other FSW population size estimates using a Bayesian mixed effects model.

Overall, we agree with the reviewer's comment that relying on data from different designs is not the ideal analysis, but we believe that it is the best approach using available data given known limitations identifying women who sell sex in household sampling frames and questionnaires. We have noted this in the limitations section of the discussion (lines 311-316):

> We have the least confidence in our estimates for the FSW risk group. As well as having the smallest sample sizes, our transactional sex estimates do not overcome the difficulties of sampling hard to reach groups. We inherent any limitations of the national FSW estimates which we adjust our estimates of transactional sex to match. Furthermore, we do not consider seasonal migration patterns, which may particularly affect FSW size.

Figure 1 is not clear. I recommend that the authors use a table as an alternative visualization.

In our and other previous work, figures similar to Figure 1 have been effective at concisely visualising inclusion of a large number of surveys across several countries and types (see, for example, Figure 1 of Giguère et al. (2021)), and we prefer to retain this figure. Supplementary Table B.3 provides an alternative tabular summary of the surveys analysed, with sample size broken down by age group. We have extended the caption of Figure 1 (proceeding line 170) to draw attention to this alternative presentation:

> **Fig 1.** Surveys used in our analysis by year, survey type, sample size, and whether the survey included a question about transactional sex. Details of included surveys are in Supplementary Table B.3.

Editorial comments

> *Please send a completed Competing Interests' statement, including any COIs declared by your co-authors. If you have no competing interests to declare, please state: "The authors have declared that no competing interests exist". Otherwise please declare all competing interests beginning with the statement "I have read the journal's policy and the authors of this manuscript have the following competing interests:*

The competing interests' statement has been updated to the following:

> I have read the journal's policy and the authors of this manuscript have the following competing interests: KAR was supported through a consultancy via UNAIDS to conduct an early version of this work; LZ, IW, MM, and CB are employees of UNAIDS; JWE is a member of the editorial board for PLOS Global Public Health; other authors have declared that no competing interests exist.

> *Please amend your detailed Financial Disclosure statement. This is published with the article. It must therefore be completed in full sentences and contain the exact wording you wish to be published. State what role the funders took in the study. If the funders had no role in your study, please state: "The funders had no role in study design, data collection and analysis, decision to publish, or preparation of the manuscript." If any authors received a salary from any of your funders, please state which authors and which funders. If you did not receive any funding for this study, please simply state: "The authors received no specific funding for this work."*

The financial disclosure statement has been amended to clarify the role of the funders as following:

> AH was supported by the EPSRC Centre for Doctoral Training in Modern Statistics and Statistical Machine Learning (EP/S023151/1). AH, VKN, KAR, JWE were supported by the Bill and Melinda Gates Foundation (OPP1190661, OPP1164897). TMW was supported by Imperial College London (President's PhD Scholarship). SF was supported by the EPSRC (EP/V002910/2). JWE was supported by UNAIDS and National Institute of Allergy and Infectious Disease of the National Institutes of Health (R01AI136664). LZ, IW, MM and CB are employed by UNAIDS. This research was supported by the MRC Centre for Global Infectious Disease Analysis (MR/R015600/1), jointly funded by the UK Medical Research Council (MRC) and the UK Foreign, Commonwealth & Development Office (FCDO), under the MRC/FCDO Concordat program and is also part of the EDCTP2 programme supported by the European Union.
>
> LZ, IW, MM, and CB are employed by UNAIDS, who also partially funded this study. LZ, IW, MM, and CB contributed to the design of the study, revision of the manuscript, and approved the final manuscript for submission. Findings and conclusions in this manuscript are those of the authors and do not necessarily represent the official position of the funding agencies.

> *Please ensure that the funders and grant numbers match between the Financial Disclosure field and the Funding Information tab in your submission form. Note that the funders must be provided in the same order in both places as well.*

The financial disclosure fields and funding information tab have now been aligned.

> *Please provide separate figure files in .tif or .eps format only and remove any figures embedded in your manuscript file. Please also ensure that all files are under our size limit of 10MB. For more information about figure files please see our guidelines: https://journals.plos.org/globalpublichealth/s/figures, https://journals.plos.org/globalpublichealth/s/figures#loc-file-requirements*

Separate figure files have been provided as requested.

> *Figure 2: please (a) provide a direct link to the base layer of the map (i.e., the country or region border shape) and ensure this is also included in the figure legend; and (b) provide a link to the terms of use / license information for the base layer image or shapefile. We cannot publish proprietary or copyrighted maps (e.g. Google Maps, Mapquest) and the terms of use for your map base layer must be compatible with our CC-BY 4.0 license. Note: if you created*

the map in a software program like R or ArcGIS, please locate and indicate the source of the basemap shapefile onto which data has been plotted. If your map was obtained from a copyrighted source please amend the figure so that the base map used is from an openly available source. Alternatively, please provide explicit written permission from the copyright holder granting you the right to publish the material under our CC-BY 4.0 license. Please note that the following CC BY licenses are compatible with PLOS license: CC BY 4.0, CC BY 2.0 and CC BY 3.0, meanwhile such licenses as CC BY-ND 3.0 and others are not compatible due to additional restrictions. If you are unsure whether you can use a map or not, please do reach out and we will be able to help you. The following websites are good examples of where you can source open access or public domain maps: U.S. Geological Survey (USGS) - All maps are in the public domain. (http://www.usgs.gov), PlaniGlobe - All maps are published under a Creative Commons license so please cite "PlaniGlobe, http://www.planiglobe.com, CC BY 2.0" in the image credit after the caption. (http://www.planiglobe.com/?lang=enl), Natural Earth - All maps are public domain. (http://www.naturalearthdata.com/about/terms-of-use/)

We attach an email from UNAIDS confirming approval for use of these subnational boundary shapefiles.

References

Abdul-Quader, Abu S, Andrew L Baughman, and Wolfgang Hladik. 2014. "Estimating the Size of Key Populations: Current Status and Future Possibilities." *Current Opinion in HIV and AIDS* 9 (2): 107–14.

Giguère, Katia, Jeffrey W Eaton, Kimberly Marsh, Leigh F Johnson, Cheryl C Johnson, Eboi Ehui, Andreas Jahn, et al. 2021. "Trends in Knowledge of HIV Status and Efficiency of HIV Testing Services in Sub-Saharan Africa, 2000–20: A Modelling Study Using Survey and HIV Testing Programme Data." *The Lancet HIV* 8 (5): e284–93.

Stevens, Oliver, Keith Sabin, Sonia Arias Garcia, Kalai Willis, Abu Abdul-Quader, Anne McIntyre, Frances Cowan, et al. 2022. "Estimating key population size, HIV prevalence, and ART coverage for sub-Saharan Africa at the national level."
